# Effects of trastuzumab and afatinib on kinase activity in gastric cancer cell lines

**DOI:** 10.1002/1878-0261.12170

**Published:** 2018-03-10

**Authors:** Simone Keller, Gwen Zwingenberger, Karolin Ebert, Jan Hasenauer, Jacqueline Wasmuth, Dieter Maier, Ivonne Haffner, Katrin Schierle, Gregor Weirich, Birgit Luber

**Affiliations:** ^1^ Institut für Allgemeine Pathologie und Pathologische Anatomie Technische Universität München Germany; ^2^ Helmholtz Zentrum München Deutsches Forschungszentrum für Gesundheit und Umwelt (GmbH) Institute of Computational Biology Neuherberg Germany; ^3^ Department of Mathematical Modeling of Biological Systems Center for Mathematics Technische Universität München Garching Germany; ^4^ Biomax Informatics AG Planegg Germany; ^5^ Universitäres Krebszentrum Leipzig (UCCL) Germany; ^6^ Institute of Pathology Universitätsklinikum Leipzig Germany

**Keywords:** afatinib, gastric cancer, HER2, kinase activity, proteome profiler, Trastuzumab

## Abstract

The molecular mechanism of action of the HER2‐targeted antibody trastuzumab is only partially understood, and the direct effects of trastuzumab on the gastric cancer signaling network are unknown. In this study, we compared the molecular effect of trastuzumab and the HER kinase inhibitor afatinib on the receptor tyrosine kinase (RTK) network and the downstream‐acting intracellular kinases in gastric cancer cell lines. The molecular effects of trastuzumab and afatinib on the phosphorylation of 49 RTKs and 43 intracellular kinase phosphorylation sites were investigated in three gastric cancer cell lines (NCI‐N87, MKN1, and MKN7) using proteome profiling. To evaluate these effects, data were analyzed using mixed models and clustering. Moreover, proliferation assays were performed. Our comprehensive quantitative analysis of kinase activity in gastric cancer cell lines indicates that trastuzumab and afatinib selectively influenced the HER family RTKs. The effects of trastuzumab differed between cell lines, depending on the presence of activated HER2. The effects of trastuzumab monotherapy were not transduced to the intracellular kinase network. Afatinib alone or in combination with trastuzumab influenced HER kinases in all cell lines; that is, the effects of monotherapy and combination therapy were transduced to the intracellular kinase network. These results were confirmed by proliferation analysis. Additionally, the MET‐amplified cell line Hs746T was identified as afatinib nonresponder. The dependence of the effect of trastuzumab on the presence of activated HER2 might explain the clinical nonresponse of some patients who are routinely tested for HER2 expression and gene amplification in the clinic but not for HER2 activation. The consistent effects of afatinib on HER RTKs and downstream kinase activation suggest that afatinib might be an effective candidate in the future treatment of patients with gastric cancer irrespective of the presence of activated HER2. However, MET amplification should be taken into account as potential resistance factor.

**Abbreviations**
A: afatinib
ADCC: antibody‐dependent cell‐mediated cytotoxicity
Ctrl: control
DMSO: dimethyl sulfoxide
EGFR: epidermal growth factor receptor
HER: human epidermal growth factor receptor
IHC: immunohistochemistry
ref. protein: reference protein
RTK: receptor tyrosine kinase
T: trastuzumab

## 1 Introduction

Gastric cancer is the fifth most common malignancy in the world and the third leading cause of cancer‐related death (Ferlay *et al*., 2015). Despite advances in treatment, the prognosis of patients is still poor, which may be due, at least in part, to the molecular complexity and heterogeneity of gastric cancer (Lordick and Janjigian, 2016). Improving the quality of life and survival of patients suffering from this disease is still challenging and requires the standardization of treatment and a better understanding of the effects of tumor biology on therapeutic outcome (Lordick *et al*., 2014).

The human epidermal growth factor receptor (HER) family of membrane‐bound RTKs has been suggested as a therapeutic target for a long time. In clinical practice, HER2 is the only HER family member that is currently used as a therapeutic target in patients with gastric cancer. In the Trastuzumab for Gastric Cancer (ToGA) trial, trastuzumab combined with chemotherapy improved the overall survival of HER2‐positive patients with advanced gastric or gastroesophageal junction cancer by roughly 3 months (Bang *et al*., 2010). These results led to the approval of trastuzumab, which is now the standard of care in combination with platin–fluoropyrimidine chemotherapy for patients with HER2‐positive recurrent or metastatic gastric cancers.

However, resistance mechanisms such as bypass track signaling by activated HER3 or MET were observed, leading to the maintenance of HER2 downstream signaling despite upstream HER2 inhibition (Arteaga and Engelman, 2014). Lung cancer cell lines treated with EGFR TKI gefitinib developed acquired resistance due to MET overexpression. This resistance was reversed by MET signaling inhibition (Engelman *et al*., 2007; Turke *et al*., 2010). Thus, MET overexpression is a potential resistance factor regarding targeted inhibition of HER family members. Intracellular kinases downstream of the RTKs, in the PI3K/AKT pathway, for instance, can be aberrantly activated as a result of somatic mutations and lead to therapy resistance (Arteaga and Engelman, 2014). In one study, *PIK3CA* mutations were associated with the reduced efficacy of trastuzumab‐ and lapatinib‐based therapies in patients with breast cancer (Majewski *et al*., 2015). In a different study, *PIK3CA* mutations or low PTEN expression was associated with reduced progression‐free survival in trastuzumab‐treated patients with breast cancer (Berns *et al*., 2007).

To increase the efficacy of treatment, combinations of trastuzumab with other targeted therapies are being investigated. A phase IIa trial studied the effect of the HER2‐targeted antibody pertuzumab in combination with trastuzumab and chemotherapy in patients with HER2‐positive, advanced gastric cancer or cancer of the gastroesophageal junction. This trial was the basis for the ongoing phase III JACOB study, in which pertuzumab in combination with trastuzumab, fluoropyrimidine, and cisplatin is tested in HER2‐positive metastatic gastric and gastroesophageal junction cancer (NCT01774786) (Kang *et al*., 2014).

In addition to combination therapies, the development of the dual EGFR/HER2 small molecule inhibitor lapatinib allows the simultaneous inhibition of EGFR and HER2 with a single drug. However, lapatinib was not successful in the treatment of advanced gastric cancer (Hecht *et al*., 2016; Lorenzen *et al*., 2015; Satoh *et al*., 2014). Other attempts to block HER family‐dependent signaling were also unsuccessful: The addition of the EGFR‐targeted antibody cetuximab to capecitabine or cisplatin also failed to improve patient outcome in the phase III EXPAND trial (Lordick *et al*., 2013). The combination of the anti‐EGFR antibody panitumumab with chemotherapy did not improve the overall survival of patients in the phase III REAL3 trial either (Waddell *et al*., 2013). Furthermore, the small molecule inhibitor erlotinib did not improve patient outcome in the SWOG 0127 trial (Dragovich *et al*., 2006).

Simultaneous blocking of the entire HER family might improve therapeutic efficacy. Studies testing the pan‐HER family‐blocker afatinib in patients with gastric cancer are currently ongoing. In patients with HER2‐overexpressing tumors, the maximum tolerated dose of afatinib in combination with trastuzumab is evaluated (Boehringer Ingelheim 2017). Additionally, three phase II studies investigate the combination of afatinib with chemotherapy. One study analyzes afatinib in combination with cisplatin and 5‐fluorouracil (FU) as a first‐line therapy in advanced gastric cancer (Hellenic Cooperative Oncology Group 2017). The combination of paclitaxel and afatinib is currently being examined in patients with EGFR‐positive gastric tumors as a second‐line therapy (Yonsei University 2017), and another phase II study investigates afatinib in combination with paclitaxel and trastuzumab in refractory esophagogastric cancer (Memorial Sloan Kettering Cancer Center 2017).

The multitude of drug candidates and the large percentage of failed clinical trials clearly indicate that the mechanism of action for the therapeutic intervention of HER signaling is still incompletely understood. In this study, we set out to close some of these gaps for trastuzumab and afatinib. Therefore, using proteome profiler analysis, we analyzed the effect of trastuzumab and afatinib treatment on the gastric cancer RTK network and the downstream‐acting intracellular kinases in gastric cancer cell lines. In our cell culture models, we focused on the direct molecular effects of trastuzumab and did not investigate the antibody‐dependent cell‐mediated cytotoxicity (ADCC) effect which occurs additionally *in vivo*. With this study, we filled some of the existing gaps in knowledge and provide quantification of the phosphoprotein abundance in three gastric cancer cell lines. Analysis of the experimental data, which was performed using clustering methods, allows us to assess the response patterns, mode‐of‐action mechanism, and resistance mechanism.

## 2 Materials and methods

### 2.1 Cell lines and cell culture conditions

The human gastric cancer cell lines MKN1, MKN7, and NCI‐N87 were used. MKN1 and MKN7 cells were supplied by the Cell Bank RIKEN BioResource Center (Tsukuba, Japan). NCI‐N87 cells were obtained from the ATCC Cell Biology Collection (LGC Standards GmbH, Wesel, Germany).

MKN1, MKN7, and NCI‐N87 were grown in RPMI 1640 medium (Thermo Fisher Scientific, Darmstadt, Germany) supplemented with 2 mm L‐glutamine (Thermo Fisher Scientific), 10% fetal bovine serum (FBS) Sera Plus (PAN Biotech, Aidenbach, Germany), and 0.5% penicillin/streptomycin (Thermo Fisher Scientific). Hs746T cells were grown in Dulbecco's modified Eagle's medium (DMEM) with GlutaMAX™ I, supplemented with 10% fetal bovine serum (FBS) Sera Plus (PAN Biotech, Aidenbach, Germany), and 0.5% penicillin/streptomycin (Thermo Fisher Scientific). After thawing, the absence of mycoplasma was confirmed routinely in conditioned medium with the PCR Mycoplasma Test Kit I/C (Promokine, Heidelberg, Germany), according to the manufacturer's instructions. The identity of each cell line was verified via short tandem repeat (STR) analysis of nine independent PCR systems by Eurofins Genomics (Ebersberg, Germany).

### 2.2 Western blot analyses

Western blot analyses were performed as reported earlier (Heindl *et al*., 2012; Kneissl *et al*., 2012). The cells were treated for the indicated times with 5 μg·mL^−1^ trastuzumab (Herceptin, Roche, Penzberg, Germany) and/or 0.001, 0.01, 0.5, or 1 μm afatinib (BIBW2992, Selleckchem distributed by Biozol, Eching, Germany). Afatinib was dissolved in dimethyl sulfoxide (DMSO). Control experiments with DMSO were also carried out (Fig. S2 and Fig. 9).

A total of 15–25 μg of total protein was loaded. The following antibodies were used: anti‐EGFR (Cell Signaling Technology (CST), distributed by New England Biolabs in Frankfurt, Germany; #2232; dilution 1 : 1000 in 5% BSA TBS‐T), anti‐pEGFR Y1068 (Life Technologies, Darmstadt, Germany; # 44788G; dilution 1 : 2000 in 5% milk TBS‐T), anti‐HER2 (Cell Signaling Technology (CST), distributed by New England Biolabs in Frankfurt, Germany; #2165; dilution 1 : 1000 in 5% BSA TBS‐T), anti‐pHER2 Y1221/2 (Cell Signaling Technology (CST), distributed by New England Biolabs in Frankfurt, Germany; # 2249; dilution 1 : 1000 in 5% BSA TBS‐T), anti‐pHER3 Y1289 (CST; # 4791; dilution 1 : 1000 in 5% BSA TBS‐T), anti‐α‐tubulin (Sigma‐Aldrich, Steinheim, Germany; # T9026; dilution 1 : 10 000 in 5% milk TBS‐T), anti‐β‐actin (Sigma‐Aldrich; A1978; dilution 1 : 5000 in 5% milk TBS‐T), anti‐mouse IgG (GE Healthcare, distributed by VWR in Ismaning, Germany; # NA931; dilution 1 : 10 000 in 5% milk TBS‐T), and anti‐rabbit IgG (CST; # 7074; dilution 1 : 2000 in TBS‐T). For quantification, the signals were densitometrically analyzed using ImageJ 1.44p Software (National Institutes of Health, Bethesda, Maryland, USA). For statistical analyses of western blot analyses, pairwise comparisons of samples were performed by two‐sided Welch's *t*‐tests. One‐sample *t*‐test was used to test the ratio of treated to untreated samples. Data analyses were performed on an explorative significance level of 0.05 using the statistical software IBM SPSS Statistics 23 (IBM, Armonk, NY, USA).

### 2.3 Proteome profiler analysis

The Human Phospho‐RTK (receptor tyrosine kinase) Array Kit and the Human Phospho‐Kinase Array Kit (#ARY001B and #ARY003B, R&D Systems, Wiesbaden‐Nordenstadt, Germany) were used to analyze the influences of different treatments on kinase phosphorylation in three gastric cancer cell lines. The changes in the activation of kinases due to the inhibition of HER receptors by trastuzumab and/or afatinib and the stimulation of EGFR and HER3 by epidermal growth factor (EGF) and neuregulin 1 (NRG1) were analyzed. Using the Phospho‐RTK Array, the level of tyrosine phosphorylation of 49 receptor tyrosine kinases can be analyzed, whereas the Phospho‐Kinase Array detects the levels of 43 specific kinase phosphorylation sites and two related total proteins. Each specific antibody is spotted in duplicate on the membranes, which also include appropriate controls.

Experiments were performed in triplicate. For the preparation of total protein extracts, 2 × 10^4^ cells·cm^−2^ for MKN1 and MKN7 and 2.4 × 10^4^ cells·cm^−2^ for NCI‐N87 were seeded. After 24 h, the cells were treated with 5 μg·mL^−1^ trastuzumab, 0.5 μm afatinib, 5 ng·mL^−1^ EGF, or 20 ng·mL^−1^ NRG1 for 5 or 20 min, as indicated in each single experiment. The preparation of cellular extracts and the proteome profiling were carried out according to the manufacturer's instructions. Briefly, lysates were diluted in blocking buffer with a total protein amount of 300 μg per array in the Phospho‐RTK Array and 400 μg in the Phospho‐Kinase Array and then incubated overnight with preblocked membranes. After several washing steps, the membranes were incubated in the provided detection antibody cocktail for 2 h, followed again by washing and incubation in streptavidin–horseradish peroxidase (HRP) for 30 min. Unbound HRP antibody was washed out before the signals were analyzed by a chemiluminescent substrate and detected by X‐ray arrays. For quantification, the signals were densitometrically analyzed using ImageJ.

### 2.4 Statistical analysis of proteome profiler data

The measured proteome profiler data were log_2_‐transformed to improve the normality and analyzed using the MATLAB statistical toolbox. For each phosphorylated protein, a linear mixed model was inferred to evaluate the treatment response, the batch effect, and the noise level. This yielded fold changes and *P*‐values for each phosphorylated protein and each pair of treatments. The *P*‐values were adjusted using the Bonferroni‐Holm correction for multiple testing. For visualization purposes, the adjusted *P*‐values were categorized (≤ 0.001; 0.001‐0.01; 0.01‐0.05), and only significant changes with (i) a fold change > 1.5 and (ii) an average control greater than twice the array internal negative control were considered.

### 2.5 Assessment of HER2 status

The HER2 status in the cell lines MKN1, MKN7, and NCI‐N87 was evaluated by immunohistochemistry (IHC). Cells were seeded with a density of 2 × 10^4^ cells·cm^−2^ for MKN1 and MKN7, and 2.4 × 10^4^ cells·cm^−2^ for NCI‐N87. After 24 h, the cells were washed, scraped off, and fixed with 4% formalin. The next day, the cells were centrifuged and the pellet was resuspended in a smaller volume of formalin. Richard‐Allan Scientific™ HistoGel™ was then added to the cell pellet. After an appropriate cooling time, the mixture block was transferred into a histocassette and embedded in paraffin. Slices were prepared, and the antibody anti‐HER‐2/neu (Roche Penzberg, Germany; #4B5) was used for IHC.

### 2.6 WST‑1 cell proliferation assay

To analyze the effects of trastuzumab and afatinib on cell proliferation, the WST‐1 cell proliferation assay (Roche Diagnostics, Mannheim, Germany) was performed according to the manufacturer's instructions. All samples were analyzed in four technical replicates. Cells were seeded at densities between 4.5 × 10^3^ and 1.2 × 10^4^ cells·cm^−2^. The following day, the cells were treated with 5 μg·mL^−1^ trastuzumab, 0.5 μm afatinib, trastuzumab solvent control (EMA 2004), or afatinib solvent control (DMSO). The trastuzumab solvent was prepared dissolving 3.36 mg L‐histidine HCl, 2.16 mg L‐histidine, 136.2 mg trehalose dehydrate, and 0.6 mg polysorbate 20 in 7.2 mL sterile water. After 72 h of incubation, the WST‐1 reagent was added. The absorbance was measured with the Asys Expert Plus microplate reader 1.5 h after the WST‐1 reagent addition. Three biological replicates were analyzed. The data are presented as the means ± standard deviation (SD). One‐sample *t*‐test was used to test the activity ratio of treated samples to untreated samples against a reference value of 100%, which indicates equality of activity. Data analyses were performed on an explorative significance level of 0.05 using the statistical software IBM SPSS Statistics 23 (IBM, Armonk, NY, USA).

## 3 Results

### 3.1 Establishment of experimental conditions for proteome profiler analysis

To investigate the effect of trastuzumab on cell signaling in gastric cancer cell lines, we assessed the phosphorylation status of 49 RTKs and 37 intracellular kinases (for which 43 kinase phosphorylation sites were considered). In addition to assessing the effects of trastuzumab treatment alone, we assessed the effects of combination treatments of trastuzumab with other HER inhibitors. In a screening setup, we tested two antibodies (cetuximab, pertuzumab) and two tyrosine kinase inhibitors (afatinib, lapatinib) for HER2 and EGFR inhibition efficacy by western blot (data not shown). The establishment of experimental conditions was performed in NCI‐N87 cells, as this line is a known trastuzumab responder (Kim *et al*. 2008, Patel *et al*. 2009, Ko *et al*. 2015, Yokoyama *et al*. 2006, Yamashita‐Kashima *et al*. 2011, Garner *et al*. 2013). Based on the screening results, afatinib was the inhibitor of choice, as it displayed the highest inhibitory activity. The ligands EGF and NRG1 were included into the experimental setup to stimulate the phosphorylation of EGFR and HER3, respectively. As the phosphorylation of HER2 homodimers is ligand‐independent, the direct stimulation of HER2 by extracellular ligands was not feasible. The gastric cancer cell lines NCI‐N87, MKN1, and MKN7 were chosen because of their different HER2 expression levels (Forbes *et al*., 2015). The IHC reveals a strong HER2 overexpression for NCI‐N87 and for the cell line MKN7. The HER2 expression for MKN1 cells is negative. Moreover, the IHC pictures demonstrate the localization of HER2 mainly in the plasma membrane of NCI‐N87 and MKN7 cells (Fig. S1).

**Figure 1:**
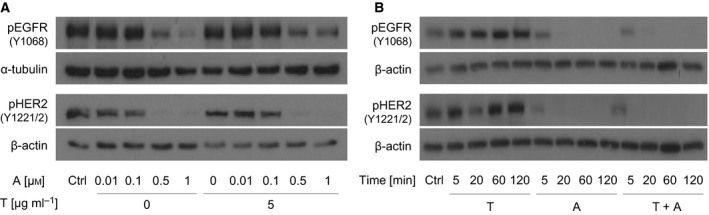
Kinetic and concentration‐dependent effects of trastuzumab and afatinib on the activation of EGFR and HER2 in NCI‐N87 cells. The levels of activated receptors were determined after treatment of NCI‐N87 cells with trastuzumab and/or afatinib by western blot analysis. Activation of the receptors was analyzed using pEGFR‐specific (Y1068) and pHER2‐specific (Y1221/2) antibodies. (A) The levels of activated receptors were determined after 5 min of treatment of NCI‐N87 cells with afatinib (0.01, 0.1, 0.5, 1 μm) and/or trastuzumab (5 μg·mL^−1^). (B) Cells were treated for 5, 20, 60, and 120 min with trastuzumab (5 μg·mL^−1^) and/or afatinib (0.5 μm). Equal loading of the lanes was confirmed by the detection of α‐tubulin or β‐actin. The depicted results are representative of three independent experiments. The results of the densitometric analysis and *P*‐values are shown in the Supplement (Fig. S2, Fig. S3, Table S1, and Table S2). A, afatinib; T, trastuzumab; Ctrl, control.

For our study, we treated the cells with 5 μg·mL^−1^ trastuzumab and 0.5 μm afatinib for 20 min. Western blot‐based analyses revealed that NCI‐N87 cells are sensitive to these drugs with this concentration regime (Fig. 1 and Fig. S3). Furthermore, the considered time is sufficient to observe a significant response (Fig. 1 and Fig. S4). EGF (5 ng·mL^−1^) and NRG1 (20 ng·mL^−1^) were used at the previously mentioned concentrations.

### 3.2 Analysis of activated receptor tyrosine kinases

A Proteome Profiler Phospho‐RTK array, detecting the tyrosine phosphorylation of 49 human RTKs simultaneously on one membrane, was performed. Of all the spotted RTKs, the only phosphorylated proteins detected in the considered gastric cancer cell lines were anaplastic lymphoma receptor tyrosine kinase (ALK), AXL receptor tyrosine kinase (AXL), epidermal growth factor receptor (EGFR), ephrin type‐B receptor 2 (Eph B2), ephrin type‐B receptor 3 (Eph B3), fibroblast growth factor receptor 3 (FGF R3), human epidermal growth factor receptor 2 (HER2), human epidermal growth factor receptor 3 (HER3), insulin‐like growth factor 1 receptor (IGF‐1R), insulin receptor (Insulin R), and receptor tyrosine kinases, such as orphan receptor 2 (ROR2), receptor‐like tyrosine kinase (RYK), and tyrosine kinase with immunoglobulin‐like and EGF‐like domains 2 (Tie‐2) (Fig. S5A, red boxes). A representative illustration of the activated RTKs for each cell line is shown in Fig. S5B,C, and D.

**Figure 2:**
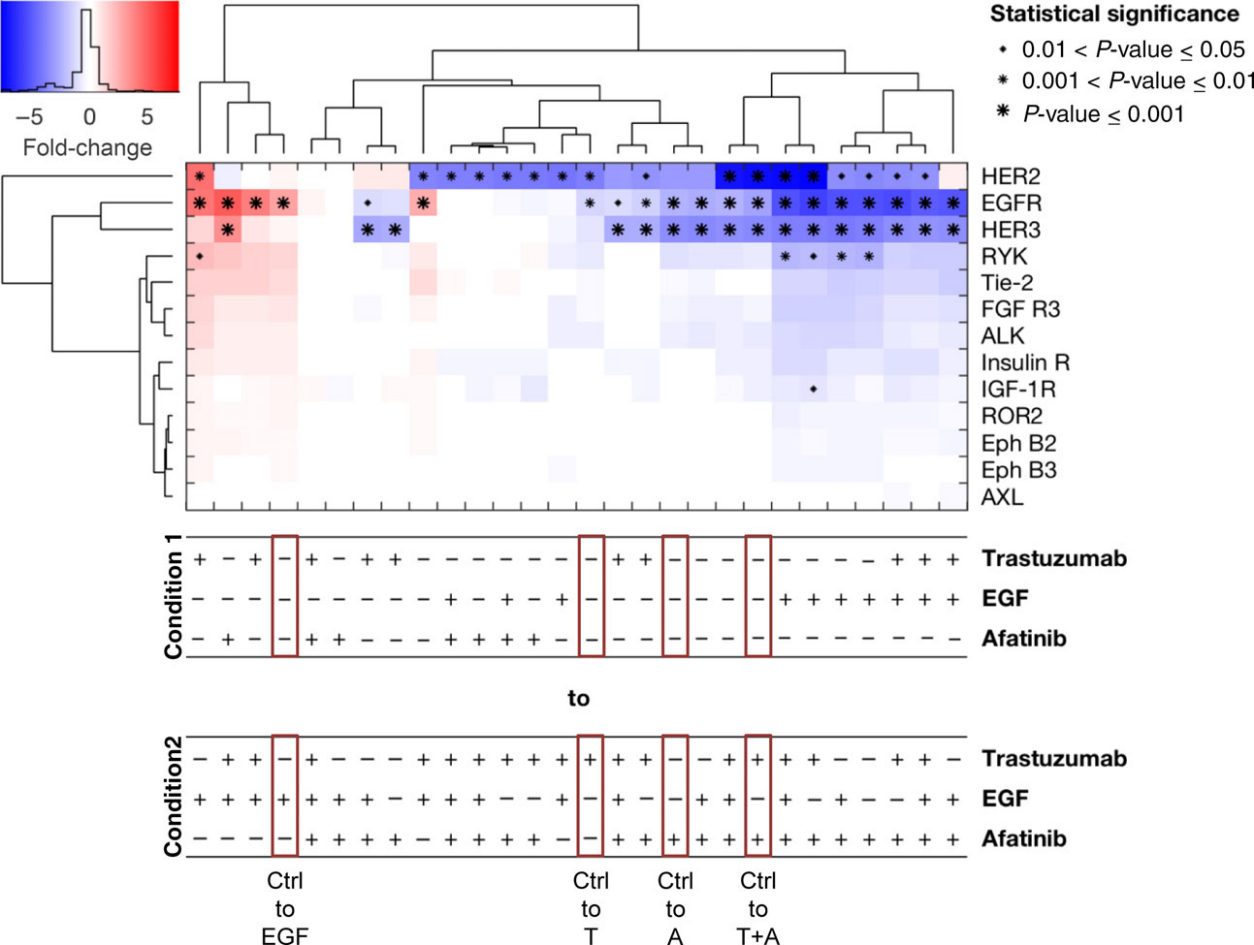
RTK Proteome Profiler analyzed by a mixed‐effect model with clustering and statistical analysis for the cell line NCI‐N87. The RTK Proteome Profiler was performed to detect the effects of trastuzumab, afatinib, and/or EGF treatment on different RTKs in NCI‐N87 cells. The cells were treated for 20 min with 5 μg·mL^−1^ trastuzumab, 0.5 μm afatinib, or 5 ng·mL^−1^ EGF as well as in combination with each other. The phosphorylation levels were quantified using densitometric analysis; a linear mixture modeling was inferred. In the cluster analysis, the x‐fold change of each activated protein is shown. Samples were clustered due to the similarity of the activated proteins and treatment conditions. Significant effects between different treatment conditions are indicated by (*) with increasing size (0.01 < *P*‐value ≤ 0.05, 0.001 < *P*‐value ≤ 0.01, and *P*‐value ≤ 0.001). A, afatinib; T, trastuzumab; Ctrl, control.

In the cell line NCI‐N87, the phosphorylation of EGFR, HER2, HER3, FGF R3, Insulin R, IGF‐1R, ALK, and RYK was detected in untreated cells (Fig. S5B). Trastuzumab treatment resulted in a reduction in phosphorylated HER2 (pHER2) and phosphorylated EGFR (pEGFR) and a slight, nonsignificant decrease in phosphorylated HER3 (pHER3) in comparison with no treatment (Fig. 2). Cells treated with afatinib showed a decrease in pEGFR and pHER3 and a slight, nonsignificant decrease in pHER2 compared to the untreated cells. The combination of trastuzumab and afatinib resulted in a strong decrease in EGFR, HER2 and HER3 phosphorylation. Thus, we observed an additional effect of trastuzumab in combination with afatinib on pHER2 in comparison with afatinib mono‐treatment. The expression of EGFR and HER2 was not altered under trastuzumab and/or afatinib treatment (Fig. S8). EGFR phosphorylation was increased after treatment with its ligand EGF. The decrease in pEGFR by trastuzumab plus/or afatinib was more pronounced in the EGF‐treated cells compared to the untreated cells (Fig. 2). The western blot shown in Fig. 1B demonstrates only slight reduction in pHER2 and no reduction in pEGFR after 20 min of treatment. This discrepancy between western blot and proteome profiler might be due to the different antibodies used. Specific antibodies used for western blot detect only one phosphorylation site, whereas pan‐antibodies used for proteome profiler detect several phosphorylation sites.

**Figure 3:**
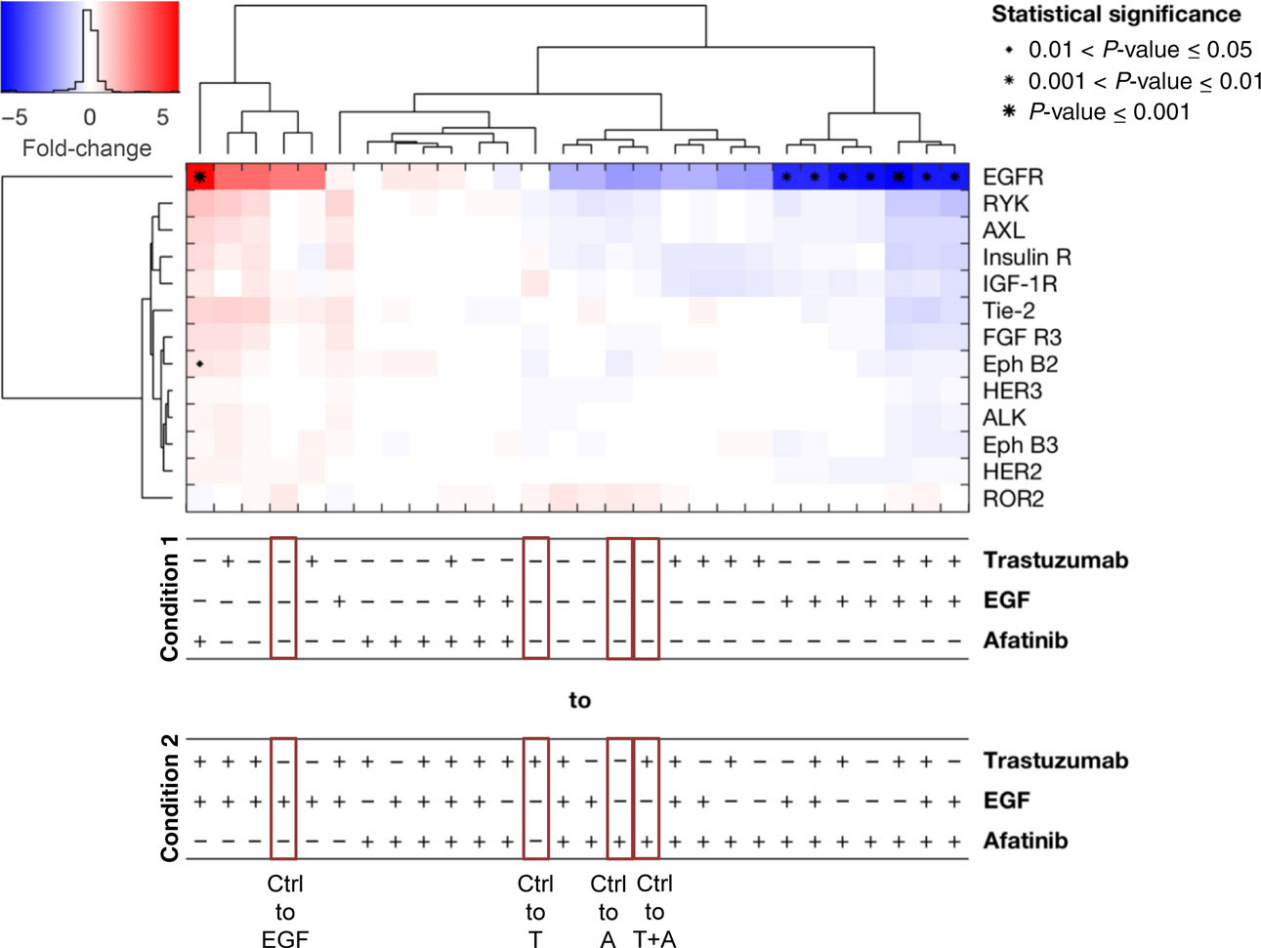
RTK Proteome Profiler analyzed by a mixed‐effect model with clustering and statistical analysis for the cell line MKN1. The RTK Proteome Profiler was performed to detect the effects of trastuzumab, afatinib, and/or EGF treatment on different RTKs in MKN1 cells. The cells were treated for 20 min with 5 μg·mL^−1^ trastuzumab, 0.5 μm afatinib, or 5 ng·mL^−1^ EGF alone as well as in combination with each other. The phosphorylation levels were quantified using densitometric analysis; a linear mixture modeling was inferred. In the cluster analysis, the x‐fold change of each activated protein is shown. Samples were clustered due to the similarity of the activated proteins and treatment conditions. Significant effects between different treatment conditions are indicated by (*) with increasing size (0.01 < *P*‐value ≤ 0.05, 0.001 < *P*‐value ≤ 0.01, and *P*‐value ≤ 0.001). A, afatinib; T, trastuzumab; Ctrl, control.

MKN1 cells showed phosphorylated EGFR, Insulin R, IGF1‐R, AXL, Tie‐2, Eph B2, Eph B3, and RYK (Fig. S5C). Interestingly, ROR2 was phosphorylated in the cell line MKN1 but not in the cell line NCI‐N87. Under treatment, the activation of EGFR changed exclusively (Fig. 3). Trastuzumab treatment had no effect in the cell line MKN1. Phosphorylation of EGFR was increased by EGF treatment and decreased by afatinib treatment (nonsignificantly). The latter was the case independent of the starting condition.

**Figure 4:**
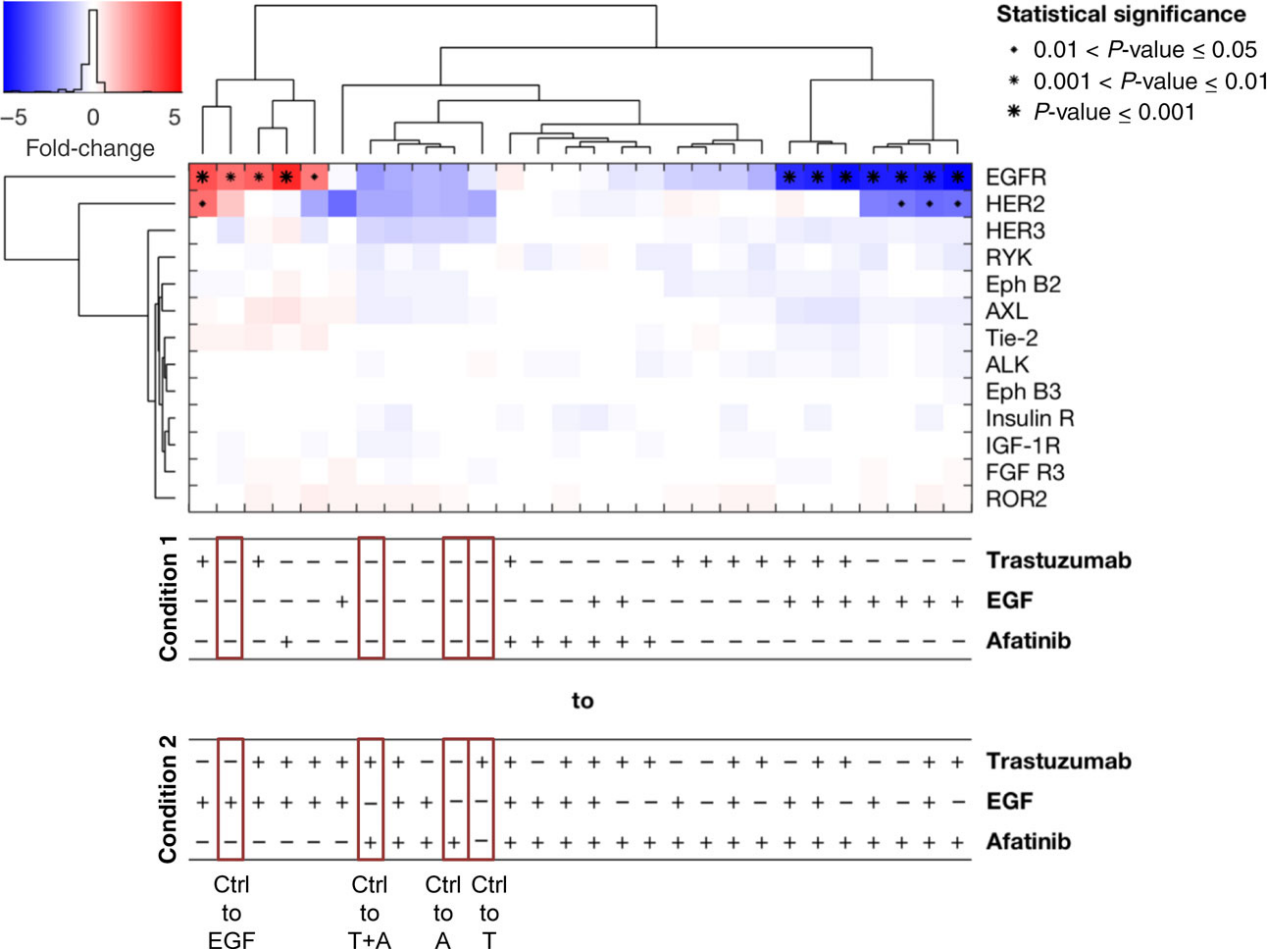
RTK Proteome Profiler analyzed by a mixed‐effect model with clustering and statistical analysis for the cell line MKN7. The RTK Proteome Profiler was performed to detect the effects of trastuzumab, afatinib, and/or EGF treatment on different RTKs in MKN7 cells. The cells were treated for 20 min with 5 μg·mL^−1^ trastuzumab, 0.5 μm afatinib, or 5 ng·mL^−1^ EGF as well as in combination with each other. The phosphorylation levels were quantified using densitometric analysis; a linear mixture modeling was inferred. In the cluster analysis, the x‐fold change of each activated protein is shown. Samples were clustered due to the similarity of the activated proteins and treatment conditions. Significant effects between the different treatment conditions are indicated by (*) with increasing size (0.01 < *P*‐value ≤ 0.05, 0.001 < *P*‐value ≤ 0.01, and *P*‐value ≤ 0.001). A, afatinib; T, trastuzumab; Ctrl, control.

The cell line MKN7 showed phosphorylated EGFR, HER2, HER3, Insulin R, IGF‐1R, and RYK. The strong activation of AXL and, as in the cell line MKN1, the phosphorylation of ROR2 (Fig. S5D) were noticeable. All treatments almost exclusively changed the phosphorylation of EGFR and HER2 (Fig. 4). Trastuzumab treatment resulted in a moderate, nonsignificant decrease in HER2 phosphorylation. Therefore, MKN7 cells showed only a slight response at the HER2 receptor level. Afatinib slightly decreased the phosphorylation of EGFR and HER2 (nonsignificantly). The combination of afatinib and trastuzumab showed a similar inhibition pattern as afatinib alone. Under treatment with the ligand EGF, the phosphorylation of EGFR was particularly increased. The difference in the inhibition of EGFR between afatinib‐treated cells and EGF‐treated cells was more pronounced than that between afatinib‐treated cells and nontreated cells. Similar to NCI‐N87, the expression of EGFR and HER2 was not altered in the cell lines MKN1 and MKN7 under trastuzumab and/or afatinib treatment (Fig. S8).

To compare the activation of EGFR and HER2 between the different cell lines directly, untreated cell lysates were analyzed on one membrane by western blot. EGFR activation was higher in NCI‐N87 cells compared to MKN7 and MKN1 cells. MKN1 and MKN7 cells showed similar EGFR activation. Regarding HER2, the activation was highest in NCI‐N87 cells, whereas MKN7 demonstrated low phosphorylation. In MKN1 cells, HER2 phosphorylation was not detectable. However, antibodies specific for one phosphorylation site were used for western blot analysis, whereas antibodies detecting several phosphorylation sites were used for RTK profiler analysis (Fig. S7).

The clustering with respect to conditions revealed the strong impact of EGF and afatinib. The analysis indicated that the inhibiting effect of afatinib treatment was consistent across conditions in all analyzed cell lines (Figs 2, 3, 4), while the effect of trastuzumab came up in the lower levels of the tree, showing that this effect was context‐specific and independent of the condition.

In summary, the only RTKs that were influenced by trastuzumab, afatinib, and EGF in the gastric cancer cell lines were the HER receptors. The effect of these different compounds varies between cell lines. In particular, under trastuzumab treatment, the effects range from a significant response (NCI‐N87) to a slight, nonsignificant response (MKN7) to a nonresponse (MKN1). Furthermore, the cell lines differ in their basal protein phosphorylation.

### 3.3 Analysis of downstream kinase activation

To extend the knowledge from receptor‐based phosphorylation status to downstream kinases, we performed a kinase proteome profiler array. In this array, 43 kinase phosphorylation sites were analyzed. All kinases were active in the analyzed cell lines (Fig. S6).

**Figure 5:**
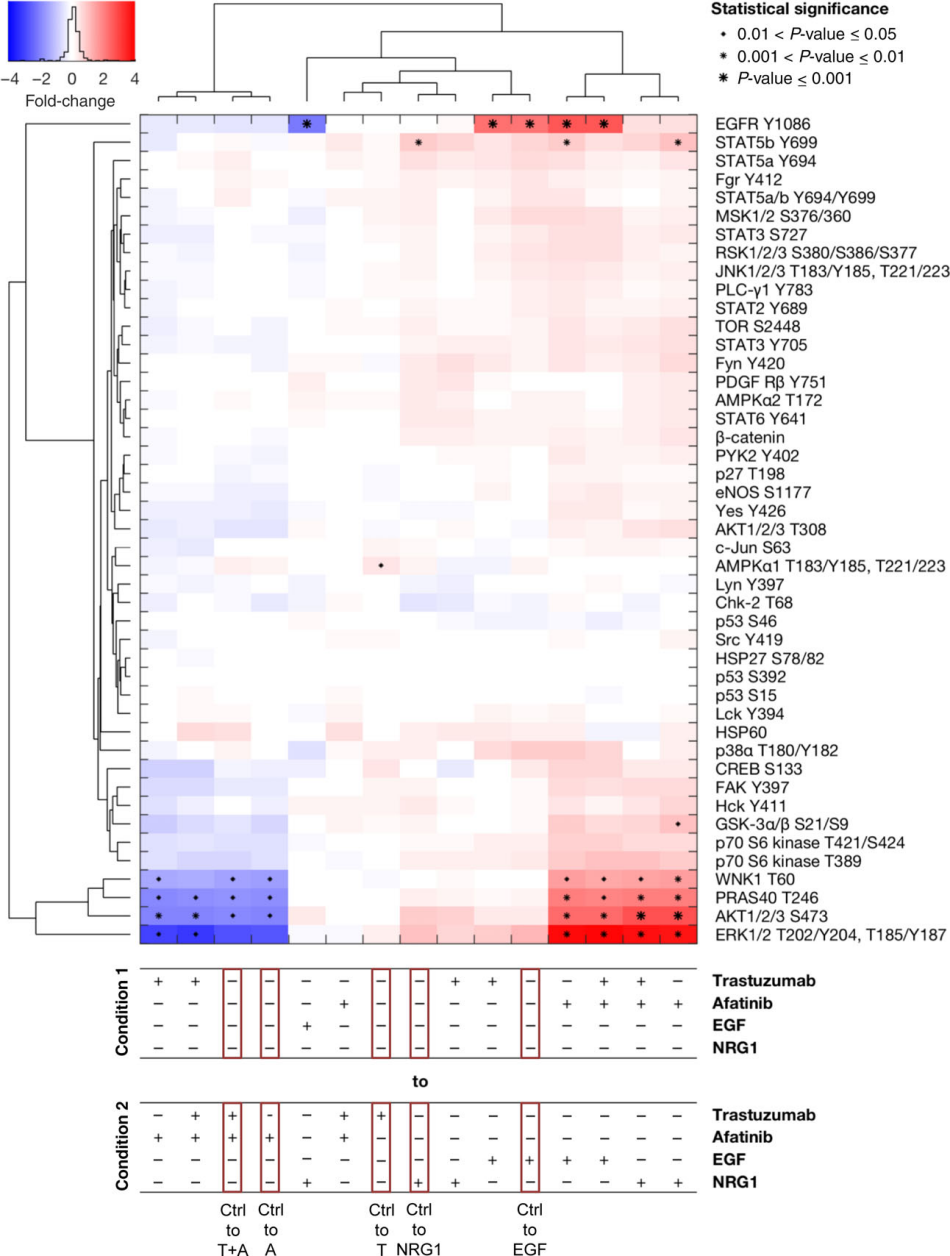
Effects of inhibition and activation of receptors on the phosphorylation of a panel of kinases in NCI‐N87 cells. The Kinase Proteome Profiler was performed to detect the effects of trastuzumab and/or afatinib treatment on downstream kinases in NCI‐N87 cells. The cells were treated for 20 min with 5 μg·mL^−1^ trastuzumab, 0.5 μm afatinib, or the combination of both drugs or were stimulated for 5 min with 5 ng·mL^−1^ EGF or 20 ng·mL^−1^ NRG1. The phosphorylation levels were quantified using densitometric analysis; a linear mixture modeling was inferred. In the cluster analysis, the x‐fold change of the activation of each included protein is shown. Samples were clustered due to the similarity of the activated proteins and treatment conditions. Significant effects between different treatment conditions are indicated by (*) with increasing size (0.01 < *P*‐value ≤ 0.05, 0.001 < *P*‐value ≤ 0.01, and *P*‐value ≤ 0.001). A, afatinib; T, trastuzumab; Ctrl, control.

In the cell line NCI‐N87, trastuzumab‐treated cells showed the same kinase phosphorylation profile as untreated cells (Fig. 5). Additionally, trastuzumab‐ plus afatinib‐treated cells showed the same phosphorylation profile as cells treated only with afatinib. Thus, the addition of trastuzumab had no measurable influence on the phosphorylation of downstream kinases and the analyzed receptors EGFR and PDGF Rβ. In contrast, NCI‐N87 cells showed decreased EGFR phosphorylation in the RTK proteome profiler array. This discrepancy can be explained by the use of pan‐antibodies in the RTK array and antibodies specific for one phosphorylation site in the kinase array. Following the stimulation of NCI‐N87 cells with EGF and the inhibition of HER receptors by afatinib, few proteins were strongly regulated. These proteins were EGFR (Y1086), ERK1/2 (T202/Y204, T185/Y187), AKT1/2/3 (S473), proline‐rich AKT substrate 40‐kDa (PRAS40 (T246)), STAT5b (Y600), and with no lysine kinase (WNK1 (T60)). EGF treatment resulted in stronger EGFR phosphorylation in comparison with no treatment. This effect was also visible for the comparison of EGF with the other treatments. NCI‐N87 cells treated with EGF or NRG1 demonstrated a small, nonsignificant increase or no increase at all in the phosphorylation of ERK1/2, AKT1/2/3, PRAS40, and WNK1 when compared to untreated cells. Looking at the comparison between EGF‐ or NRG1‐treated and afatinib‐treated cells, the phosphorylation of these kinases was increased. The reduction in ERK1/2 (nonsignificant), AKT1/2/3, PRAS40, and WNK1 phosphorylation was observed in afatinib‐treated cells compared to untreated cells. Interestingly, EGFR, the only detectable receptor in this array, did not cluster with any of the downstream kinases.

The analysis across conditions revealed that the effect of afatinib treatment was consistent across conditions, while the effect of trastuzumab treatment was context‐specific. The clustering regarding the analyzed phosphorylated proteins revealed consistent changes in ERK1/2 (T202/204, T185/Y187), AKT1/2/3 (S473), PRAS40 (T246), and WNK1 (T60) and a unique response pattern of EGFR (Y1086) in NCI‐N87 cells.

**Figure 6:**
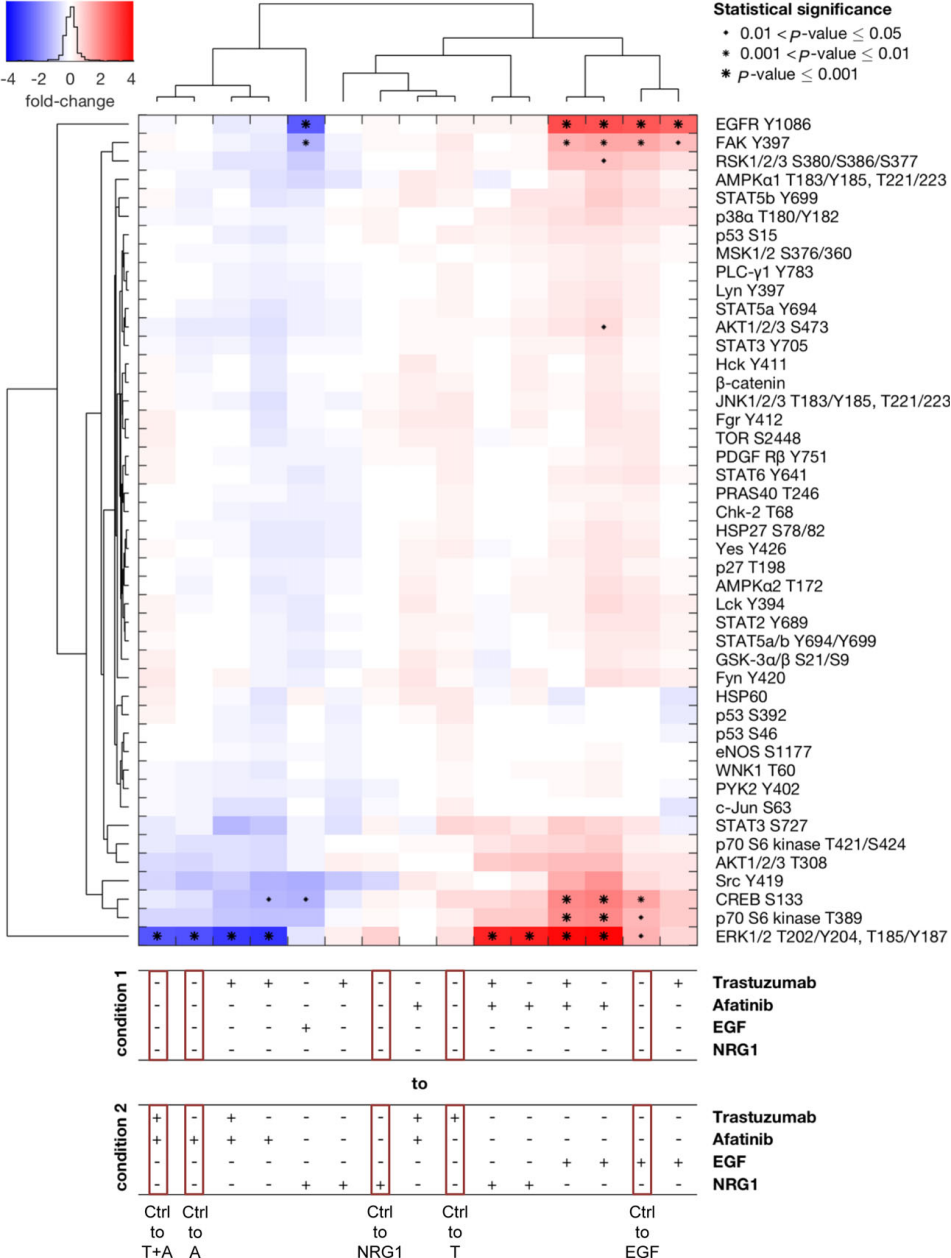
Effects of inhibition and activation of receptors on the phosphorylation of a panel of kinases in MKN1 cells. The Kinase Proteome Profiler was performed to detect the effects of trastuzumab and/or afatinib treatment on downstream kinases in MKN1 cells. The cells were treated for 20 min with 5 μg·mL^−1^ trastuzumab, 0.5 μm afatinib, or the combination of both drugs or were stimulated for 5 min with 5 ng·mL^−1^ EGF or 20 ng·mL^−1^ NRG1. The phosphorylation levels were quantified using densitometric analysis, and a linear mixture modeling was inferred. In the cluster analysis, the x‐fold change of the activation of each included protein is shown. Samples were clustered due to the similarity of the activated proteins and treatment conditions. Significant effects between different treatment conditions are indicated by (*) with increasing size (0.01 < *P*‐value ≤ 0.05, 0.001 < *P*‐value ≤ 0.01, and *P*‐value ≤ 0.001). A, afatinib; T, trastuzumab; Ctrl, control.

In the cell line MKN1, treatment with trastuzumab did not result in a measurable alteration of the phosphorylation state of the kinases, regardless of the mono‐ or combination treatment (Fig. 6). ERK1/2 (T202/Y204, T185/Y187), CREB (S133), p70 S6 kinase (T389), EGFR (Y1086), and FAK (Y397) were strongly influenced by EGF and afatinib treatment in MKN1 cells. ERK1/2 phosphorylation was induced by EGF or NRG1 treatment. The treatment of MKN1 cells with afatinib or with the combination of trastuzumab and afatinib resulted in inhibition of ERK1/2 phosphorylation. CREB and p70 S6 kinase were phosphorylated after EGF treatment but not NRG1 treatment compared to the untreated cells. EGFR and FAK phosphorylation was also significantly increased by EGF treatment. Similar to NCI‐N87 cells, EGFR did not cluster with any of the other kinases.

The clustering across conditions indicated that the inhibitory effect of afatinib treatment was the strongest and was homogenous across treatment conditions, whereas the effect of trastuzumab treatment was context‐specific. The clustering with respect to the phosphorylated proteins revealed consistent changes in ERK (T202/204, T185/Y187), p70 S6 kinase (T389), CREB (S133), and Src (Y419) in MKN1 cells. Additionally, EGF and NRG clustered separately due to the differential effects on the activation of EGFR (Y1086), FAK (Y397), and RSK1/2/3 (S380/S386/S377).

**Figure 7:**
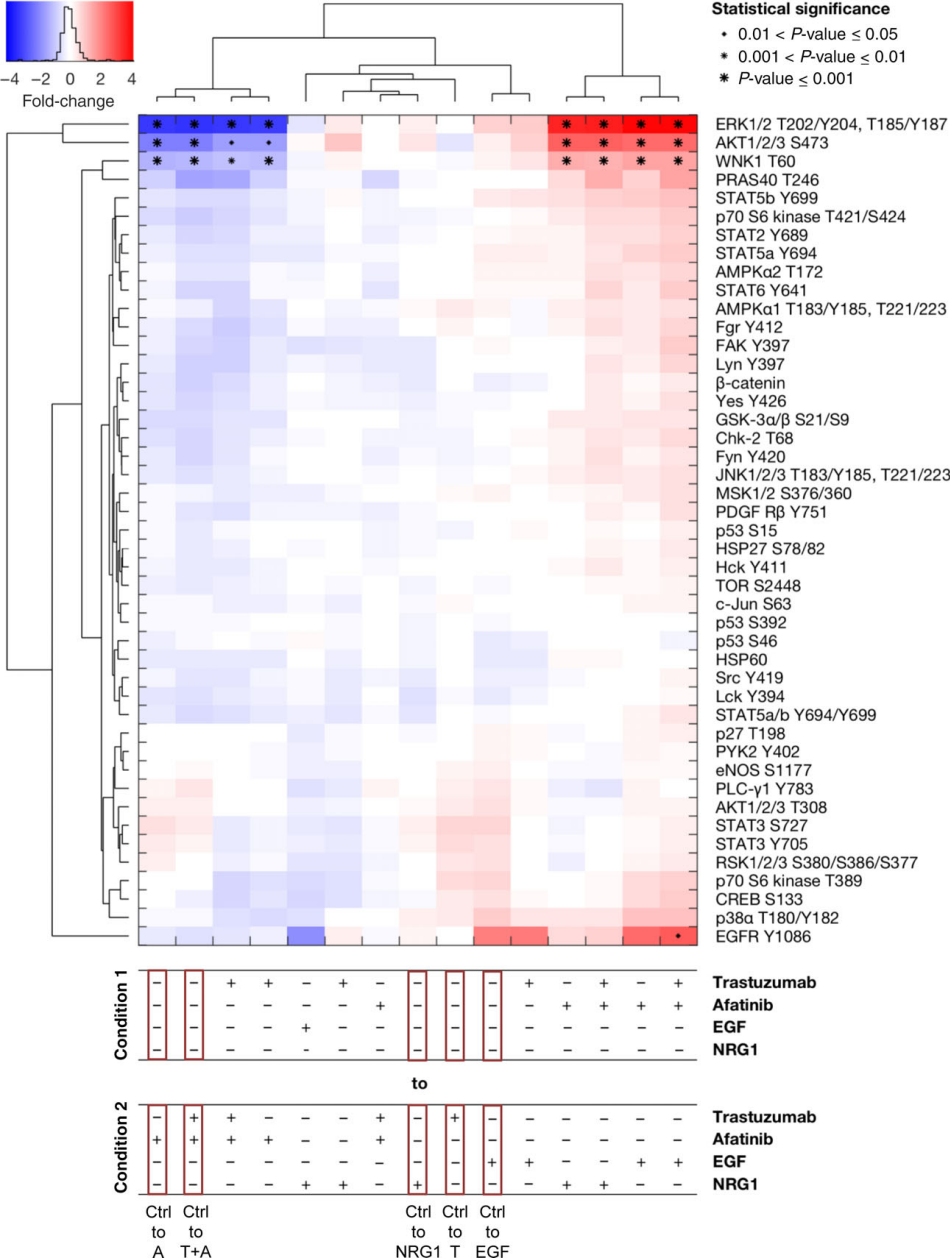
Effects of inhibition and activation of receptors on the phosphorylation of a panel of kinases in MKN7 cells. The Kinase Proteome Profiler was performed to detect the effects of trastuzumab and/or afatinib treatment on downstream kinases in MKN7 cells. The cells were treated for 20 min with 5 μg·mL^−1^ trastuzumab, 0.5 μm afatinib, or the combination of both drugs or were stimulated for 5 min with 5 ng·mL^−1^ EGF or 20 ng·mL^−1^ NRG1. The phosphorylation levels were quantified using densitometric analysis, a linear mixture modeling was inferred, and cluster analysis was performed. In the cluster analysis, the x‐fold change of the activation of each included protein is shown. Samples were clustered due to the similarity of the activated proteins and treatment conditions. Significant effects between different treatment conditions are indicated by (*) with increasing size (0.01 < *P*‐value ≤ 0.05, 0.001 < *P*‐value ≤ 0.01, and *P*‐value ≤ 0.001). A, afatinib; T, trastuzumab; Ctrl, control.

As already shown for NCI‐N87 and MKN1 cells, trastuzumab demonstrated no measurable effect on the analyzed phosphorylated kinases in MKN7 cells (Fig. 7). Moreover, the combination of trastuzumab and afatinib resulted in a similar reduction in phosphorylated kinases when compared to afatinib alone. The phosphorylation of ERK1/2 (T202/Y204, T185/Y187), AKT1/2/3 (S473), WNK1 (T60), and EGFR (Y1086) was strongly influenced by the different treatments. After EGF and NRG1 treatment, the phosphorylation of ERK1/2, AKT1/2/3, and WNK1 was unchanged compared to untreated cells. Afatinib treatment resulted in a reduced phosphorylation of ERK1/2, AKT1/2/3, and WNK1. EGFR appears to be strongly phosphorylated by EGF treatment, but this phosphorylation is not significant. In the cluster analysis, EGFR phosphorylation was completely different from the detectable downstream kinases.

The clustering with respect to the conditions revealed a strong impact of afatinib across conditions, while the effect of trastuzumab treatment was context‐specific. The clustering across analyzed phosphorylated proteins revealed consistent changes in ERK1/2 (T202/204, T185/Y187), AKT1/2/3 (S473), WNK1 (T60), and PRAS40 (T246). A unique response pattern of EGFR (Y1086) in MKN7 cells was detected.

In summary, no effect of trastuzumab treatment was observed on downstream kinase phosphorylation in the analyzed cell lines. In contrast, EGF, NRG1, and afatinib treatment changed kinase activation.

### 3.4 Trastuzumab and afatinib responsiveness of gastric cancer cell lines

The effect of trastuzumab and afatinib on the proliferation of NCI‐N87, MKN7, and MKN1 cells was analyzed in an attempt to search for differences in the phenotypic reaction of the cells to the different drugs. The Hs746T cell line was included in the analysis as a control because this cell line is a known trastuzumab nonresponder (Kneissl *et al*., 2017).

To determine the responsiveness to trastuzumab and afatinib in the panel of four human gastric cancer cell lines, cells were treated with 5 μg·mL^−1^ trastuzumab, 0.5 μm afatinib, both trastuzumab and afatinib, or the appropriate solvent control. The metabolic activity of the cell lines as a surrogate marker for cell viability was analyzed with the WST‐1 cell viability assay.

**Figure 8:**
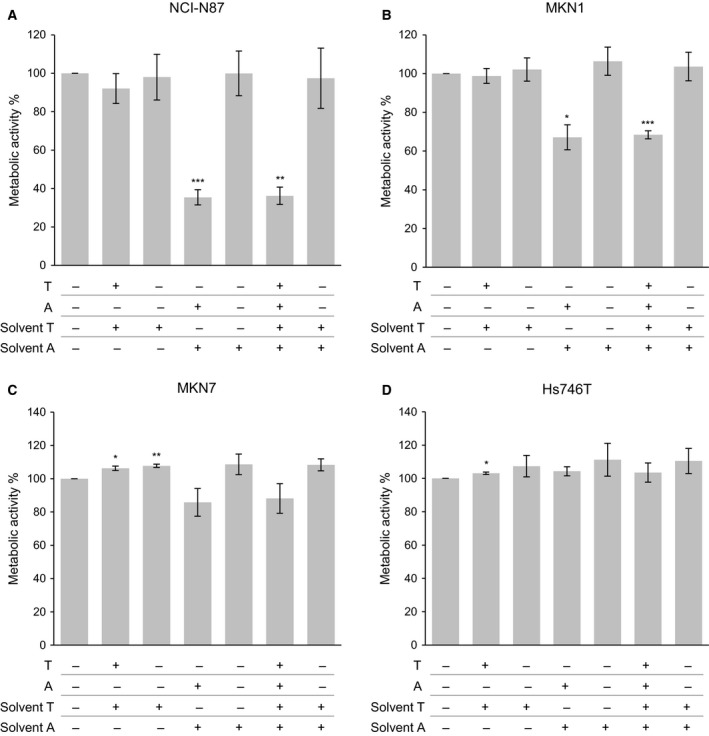
Effect of trastuzumab and afatinib on the cell proliferation of gastric cancer cell lines. The cell lines NCI‐N87, MKN1, MKN7, and Hs746T were treated for 72 h with trastuzumab (T) (5 μg·mL^−1^), afatinib (A) (0.5 μm), trastuzumab (5 μg·mL^−1^) plus afatinib (0.5 μm) (T + A), trastuzumab solvent, afatinib solvent, or trastuzumab plus afatinib solvent. The metabolic activity was determined via WST‐1 cell proliferation assay. The mean value of at least three independent experiments is shown. Significant effects compared to untreated control cells (Ctrl) are indicated by *0.01 < *P*‐value ≤ 0.05, **0.001 < *P*‐value ≤ 0.01, or ***≤ 0.001 (one‐sample *t*‐test).

Trastuzumab treatment resulted in slightly decreased (not significant) NCI‐N87 cell proliferation (Fig. 8 and Table S5). MKN1 cells demonstrated proliferation similar to that of untreated control cells, whereas MKN7 and Hs746T cells showed a slightly increased proliferation after trastuzumab treatment. However, treatment with trastuzumab solvent also resulted in a slightly increased proliferation in MKN7 and Hs746T cells. Afatinib treatment markedly decreased the proliferation to 35%, 65%, and 85% in NCI‐N87, MKN1, and MKN7 cells, respectively. In contrast, afatinib treatment did not decrease Hs746T cell proliferation. No differences in proliferation were observed between afatinib‐ and trastuzumab‐ plus afatinib‐treated NCI‐N87, MKN1, MKN7, and Hs746T cells.

**Figure 9:**
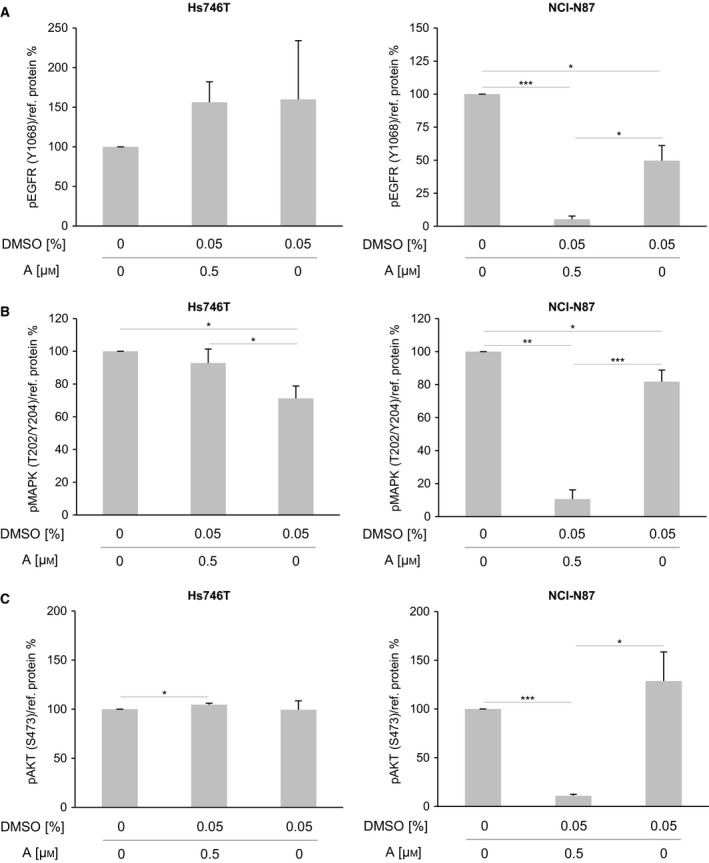
Effect of afatinib on the activation of EGFR, MAPK, and AKT in Hs746T and NCI‐N87 cells. The levels of activated receptors were determined by western blot analysis with pEGFR‐specific (Y1068), pMAPK‐specific (T202/Y204), and pAKT‐specific (S473) antibodies after a 5‐min treatment of Hs746T or NCI‐N87 cells with afatinib (0.5 μm) or DMSO (0.05%). pEGFR analysis for NCI‐N87 cells was already shown in Fig. S1. The average phosphorylation levels were quantified using densitometric analysis and calculated in relation to the levels of the reference proteins α‐tubulin and β‐actin (+SD). Significant effects are indicated by *0.01 < *P*‐value ≤ 0.05, **0.001 < *P*‐value ≤ 0.01 or ***≤ 0.001. A, afatinib.

Furthermore, the molecular reaction of Hs746T cells to afatinib treatment was compared with that of the cell line NCI‐N87. Neither RTKs (EGFR, HER2) nor downstream kinases (MAPK, AKT) were influenced by afatinib in Hs746T, whereas all analyzed kinases in NCI‐N87 cells responded to afatinib (Fig. 9; data for HER2 not shown).

## 4 Discussion

### 4.1 Effect of trastuzumab treatment in gastric cancer cell lines

Different mechanisms of action were described for the therapeutic HER2‐directed antibody trastuzumab: direct effects via uncoupling of the ligand‐independent HER2‐containing dimers (thus reducing the phosphorylation of HER2, which can lead to the partial inhibition of downstream signaling) and indirect antitumor effects by triggering antibody‐dependent cell‐mediated cytotoxicity (ADCC) (Clynes *et al*., 2000; Cuello *et al*., 2001; Fujimoto‐Ouchi *et al*., 2007; Ghosh *et al*., 2011; Hata *et al*., 2013; Junttila *et al*., 2009; Molina *et al*., 2001; Sliwkowski *et al*., 1999; Yakes *et al*., 2002).

In the present study, we analyzed the direct molecular effects of trastuzumab on the gastric cancer signaling network in the following three gastric cancer cell lines: NCI‐N87, MKN1, and MKN7. For HER2 activation, we found different trastuzumab response patterns in the different cell lines. In the cell line NCI‐N87, showing strong expression and activation of HER2 and harboring a *HER2* amplification and *HER2* mutations (Table S4), trastuzumab reduced the activation of HER2. This result suggests that trastuzumab is able to block the dimerization of HER2 with itself (homodimerization) and with other HER receptors (heterodimerization) in this cell line. In contrast, MKN1 cells did not respond to trastuzumab treatment, most likely because of the low basal HER2 expression level and the lack of HER2 activation observed in this cell line (Ishida *et al*. 2016 and Fig. S6). MKN7 cells, which are characterized by a strong expression of HER2 and an intermediate level of HER2 activation (which is below that of NCI‐N87 cells), showed less pronounced inhibition of HER2 phosphorylation under trastuzumab treatment than NCI‐N87 cells, although both cell lines, NCI‐N87 and MKN7, harbor a *HER2* amplification (Table S4). Interestingly, MKN7 cells reacted similarly to the antibody 4D5, the murine precursor to trastuzumab, which has the same antigen‐binding fragment as trastuzumab (Carter *et al*., 1992; Lane *et al*., 2000). Possible explanations for the reduced sensitivity of MKN7 cells to both trastuzumab and 4D5 include the shedding of the extracellular trastuzumab‐binding domain of HER2, which might occur in this cell line (Molina *et al*., 2002), and the lower basal HER2 phosphorylation compared to NCI‐N87 cells.

Despite the different effects of trastuzumab on the activation of HER2 observed in the three analyzed cell lines ranging from a significant response (NCI‐N87) to a slight, nonsignificant response (MKN7) to a nonresponse (MKN1), no inhibition of the downstream signaling with respect to the kinases included in the proteome profiler screening could be detected in any of those cell lines. Insufficient treatment of the cells with trastuzumab can be excluded because a comprehensive optimization of treatment conditions was performed prior to the analysis. As reported in the literature, even under higher concentrations of trastuzumab and longer treatment durations than described here, no effects on ERK and inconsistent effects on AKT occurred in the cell lines MKN7 and NCI‐N87 (Han *et al*., 2014; Leto *et al*., 2015; Tanizaki *et al*., 2010; Wainberg *et al*., 2010; Zheng *et al*., 2014). The nonresponsiveness to trastuzumab treatment of the downstream kinases in MKN7 and NCI‐N87 cells can potentially be explained by the heterodimerization of EGFR and HER3 (Neve *et al*., 2001) or persistent HER3 signaling (Motoyama *et al*., 2002, Wheeler *et al*. 2008) compensating for HER2 inhibition.

Our analysis of the phenotype by proliferation experiments demonstrated a slight decrease in metabolic activity under trastuzumab treatment in NCI‐N87 cells. The observed effects were stronger in previous reports already showing the sensitivity of NCI‐N87 cells to trastuzumab with respect to proliferation (Kim *et al*. 2008, Patel *et al*. 2009, Ko *et al*. 2015, Yokoyama *et al*. 2006, Yamashita‐Kashima *et al*. 2011, Garner *et al*. 2013) and reduced tumor volume in NCI‐N87 xenograft models (Ko *et al*. 2015, Patel *et al*. 2009, Tanner *et al*. 2005). In MKN1 and MKN7 cells, the proliferation was unchanged after trastuzumab treatment. These observations are in line with the lacking downstream kinase response of the investigated intracellular kinases observed in these cell lines in our study.

The lack of detectable HER2 activation most likely explains the trastuzumab insensitivity of MKN1 cells. In addition, the presence of a mutation in *PIK3CA* (the gene coding for PI3‐kinase Alpha), which is associated with constitutive kinase activation, might be linked to the trastuzumab resistance of MKN1 cells (Kang *et al*., 2005) (Table S4). Furthermore, our analysis showed that the receptor ROR2 is activated in MKN1 cells. ROR2 is involved in the regulation of cellular invasiveness and cell motility and is implicated in WNT signal transduction (Green *et al*., 2014). ROR2 was found to be highly expressed in a variety of cancers, and in the majority of those, ROR2 expression was associated with more aggressive disease states, consistent with its involvement in WNT signaling (Debebe and Rathmell, 2015). We present evidence that ROR2 is also activated in the cell line MKN7. In addition to the potential HER2 shedding in this cell line, another possible cause of resistance is the presence of the activated receptor AXL. Activation of AXL is associated with the motility and proliferation of cells and with tumorigenesis, which can be reduced by the inhibition of AXL (Korshunov, 2012). Moreover, AXL can be part of a resistance bypass track mechanism, which can compensate for the inhibition of HER2 (Arteaga and Engelman, 2014) and enhance cellular survival and invasion and suppress apoptosis via the AKT pathway (Sawabu *et al*., 2007). Furthermore, AXL was described as a resistance factor for HER2 therapeutics in breast cancer (Liu *et al*., 2009).

As mentioned before, trastuzumab has an indirect antitumor effect by triggering ADCC, which was not investigated in the present study. In addition to the other experiments, significantly reduced tumor volume was shown in a xenograft model with the cell line NCI‐N87 under trastuzumab treatment (Matsui *et al*., 2005). Therefore, these indirect immunological effects of trastuzumab might be of high relevance.

### 4.2 Afatinib treatment as a therapeutic option for trastuzumab‐resistant patients with gastric cancer

Only 6‐30% of gastric cancers show *HER2* gene amplification or overexpression, and approximately half of HER2 positive cancers do not respond to trastuzumab treatment (reviewed by Apicella *et al*. (2017)). Therefore, there is a need for an alternative therapy for those patients who do not benefit from trastuzumab treatment or develop resistance during therapy. This issue is addressed in a phase II clinical trial investigating the effect of afatinib on tumor growth in HER2‐positive esophagogastric cancers that do not respond to trastuzumab treatment (Memorial Sloan Kettering Cancer Center 2017). The broader inhibition profile of afatinib (EGFR, HER2, HER4) compared to trastuzumab (HER2 only) provides the rationale for its use as a therapy in patients with trastuzumab‐resistant tumors (Wind *et al*., 2016). Our *in vitro* analyses showed an inhibitory effect of afatinib on the activation of AKT1/2/3, WNK1, and ERK1/2 kinases in the HER2‐positive cell line NCI‐N87. These effects were even more pronounced in MKN7 cells, which showed less HER2 activation than NCI‐N87 cells. Moreover, afatinib demonstrated inhibitory effects on the downstream kinases ERK1/2 in the cell line MKN1. This cell line shows low HER2 expression and lacks HER2 activation, as demonstrated by Wainberg *et al*. (2010) and our data. To conclude, afatinib had an inhibitory effect on downstream signaling in the presence and even in the absence of activated HER2. Whether this inhibition has an impact on the phenotypic behavior of cells was determined with proliferation assays in which NCI‐N87, MKN1, and MKN7 cells as well as Hs746T cells were treated with trastuzumab and afatinib. NCI‐N87, MKN1, and MKN7 cells responded to afatinib treatment, whereas the cell line Hs746T did not respond to afatinib. These data are consistent with the ranking of the cell lines by their sensitivity (IC50) to afatinib determined with the minimum and maximum screening concentrations of 0.0391 μm and 10 μm, respectively (Yang *et al*., 2013). NCI‐N87 gastric cancer cells were already shown previously to be sensitive to afatinib by the MTT proliferation assay (Nam *et al*., 2012) and in xenograft models (Janjigian *et al*. 2016, Leto *et al*., 2015). The combination of afatinib and trastuzumab was as effective as afatinib alone and more effective than trastuzumab alone (Janjigian *et al*. 2013). Afatinib is a potential second‐line therapy option in HER2‐positive gastric cancer and should furthermore be taken into account as a therapeutic option in HER2‐negative gastric carcinoma.

### 4.3 Potential response and resistance factors to afatinib therapy

To identify the potential response and resistance factors toward afatinib therapy, the mutation and amplification statuses of the analyzed kinases were verified in the COSMIC cell line project database (Forbes *et al*., 2015) for the cell lines NCI‐N87, MKN1, and MKN7. Except for multiple *TP53* missense mutations in all three analyzed cell lines, none of the kinases that were inhibited by afatinib were affected by genetic alterations (see Table S4). Thus, none of the proteins that were affected by afatinib treatment in this study can be considered as a potential resistance factor due to the following reasons: (1) Key downstream kinases were sensitive to afatinib treatment in all three analyzed cell lines, and no afatinib‐resistant cell line was identified by protein profiling. (2) No damaging or activating mutations in the kinases were identified.

It is currently unclear whether known resistance factors for HER‐targeted therapies including activating mutations in genes encoding K*RAS, MET,* or *PIK3CA* (Bardelli and Siena, 2010; Kneissl *et al*., 2012; van der Wekken *et al*., 2016; Wu *et al*., 2016) provide resistance to afatinib therapy. In nonsmall cell lung cancer (NSCLC), the *EGFR* V843I mutation and *MET* amplification were described as resistance‐supporting in afatinib therapy (Chen *et al*., 2013; van der Wekken *et al*., 2016). MET is overexpressed in NCI‐N87 cells (Chen *et al*., 2012; Liu *et al*., 2011), but none of the afatinib responding cell lines used in our study revealed a MET amplification (Chen *et al*., 2012; Kawakami *et al*., 2013; Liu *et al*., 2014; Smolen *et al*., 2006; Zhao *et al*., 2013).

To clarify whether an additional *MET* amplification can provide resistance to afatinib treatment in gastric cancer cell lines, we performed experiments with the Hs746T cell line. This cell line harbors both a MET amplification and a MET mutation (Zang *et al*., 2011). In Hs746T cells, neither RTKs (EGFR, HER2) nor downstream kinases (MAPK, AKT) were influenced by afatinib. Furthermore, we observed that afatinib treatment decreased the proliferation in NCI‐N87, MKN1, and MKN7 cells but not in Hs746T cells, suggesting that *MET* amplification is a potential resistance factor in gastric cancer. The higher the IC50 for afatinib sensitivity was (Yang *et al*., 2013), the higher the detected pMET (Y1234/5) level in the cell line from a panel of six analyzed gastric cancer cell lines (data not shown). In human gastric cancer, *MET* amplification ranges between 4% and 23% (Deng *et al*., 2012; Kuniyasu *et al*., 1992; Lee *et al*., 2011; Nakajima *et al*., 1999; Peng *et al*., 2014); it remains to be determined whether *MET* amplification is a clinically important resistance factor for gastric cancer patients treated with afatinib in clinical trials.

## 5 Conclusion

Our analysis of kinase activity in gastric cancer cell lines indicates that the only RTK that was influenced by trastuzumab was HER2. HER2 activation was mandatory for successful trastuzumab treatment. However, the effect of trastuzumab on HER2 was not transduced to the investigated downstream kinases. We suggest a broader screening of downstream kinases to identify potential intracellular trastuzumab‐affected kinases that are relevant in the context of gastric cancer.

To the best of our knowledge, we show for the first time the inhibiting effects of afatinib treatment in gastric cancer cell lines in a broad proteome analysis. In our panel of cell lines, HER2 activation was not mandatory for successful afatinib treatment. In contrast to trastuzumab, afatinib showed effects on the investigated downstream kinases. Further, we identified *MET* amplification as a potential resistance factor to afatinib therapy in gastric cancer cell lines. Although the results were obtained *in vitro*, they might be relevant to clinical applications. To clarify the clinical relevance, we suggest the investigation of afatinib as a potential treatment option for trastuzumab‐resistant gastric cancer patients without *MET* amplification, independent of HER2 activation.

## Author contributions

SK and GZ conceptualized the work and executed the experiments, interpreted the data, and wrote parts of the manuscript. KE executed the experiments, helped with the data interpretation, and wrote parts of the manuscript. JH mathematically analyzed the data and wrote parts of the manuscript. JW and DM helped with the data interpretation. IH and KS prepared and performed the assessment of the HER2 status. GW implemented the method for paraffin embedding of cultured cells to assess their HER2 status. BL conceptualized the work, wrote parts of the manuscript, and acts as the corresponding author.

## Supporting information


**Fig. S1.** Assessment of the HER2 status and localization.
**Fig. S2.** Effects of DMSO on the activation of EGFR and HER2 in NCI‐N87 cells.
**Fig. S3.** Concentration‐dependent effect of trastuzumab and afatinib on the activation of EGFR and HER2 in NCI‐N87 cells.
**Fig. S4.** Kinetic effect of trastuzumab and afatinib on the activation of EGFR and HER2 in NCI‐N87 cells.
**Fig. S5.** Receptor tyrosine kinases analyzed by the RTK proteome profiler.
**Fig. S6.** Phosphorylated kinases analyzed by the kinase proteome profiler.
**Fig. S7.** Comparison of EGFR and HER2 activation in NCI‐N87, MKN1, and MKN7 cells.
**Fig. S8.** Effect of trastuzumab and afatinib on the expression of EGFR and HER2 in NCI‐N87, MKN1, and MKN7 cells.
**Table S1.** Pairwise comparisons of EGFR and HER2 activation in NCI‐N87 cells (treatment and control).
**Table S2.** Pairwise comparisons of EGFR, MAPK and AKT activation in NCI‐N87 cells between various treatments.
**Table S3.** Pairwise comparisons of the EGFR and HER2 activation in NCI‐N87 cells between different stimulation times.
**Table S4.** Alterations in the cell lines NCI‐N87, MKN1 and MKN7.
**Table S5.** Pairwise comparisons of metabolic activity between trastuzumab (T), afatinib (A) and trastuzumab + afatinib (T + A) treated NCI‐N87, MKN1, MKN7 and Hs746T cells.
**Table S6.** Pairwise comparisons of EGFR, MAPK and AKT activation between treatment (afatinib) and control (DMSO) in Hs746T and NCI‐N87 cells.
