# Supplementary material for: Effects of trastuzumab and afatinib on kinase activity in gastric cancer cell lines
Source: Mol Oncol. 2018 Mar 10;12(4):441–62. doi: 10.1002/1878-0261.12170 (PMC5891041; doi:10.1002/1878-0261.12170)
Supplement: Supplementary file 1 — Fig. S1. Assessment of the HER2 status and localization. Fig. S2. Effects of DMSO on the activation of EGFR and HER2 in NCI‐N87 cells. Fig. S3. Concentration‐dependent effect of trastuzumab and afatinib on the activation of EGFR and HER2 in NCI‐N87 cells. Fig. S4. Kinetic effect of trastuzumab and afatinib on the activation of EGFR and HER2 in NCI‐N87 cells. Fig. S5. Receptor tyrosine kinases analyzed by the RTK proteome profiler. Fig. S6. Phosphorylated kinases analyzed by the kinase proteome profiler. Fig. S7. Comparison of EGFR and HER2 activation in NCI‐N87, MKN1, and MKN7 cells. Fig. S8. Effect of trastuzumab and afatinib on the expression of EGFR and HER2 in NCI‐N87, MKN1, and MKN7 cells. Table S1. Pairwise comparisons of EGFR and HER2 activation in NCI‐N87 cells (treatment and control). Table S2. Pairwise comparisons of EGFR, MAPK and AKT activation in NCI‐N87 cells between various treatments. Table S3. Pairwise comparisons of the EGFR and HER2 activation in NCI‐N87 cells between different stimulation times. Table S4. Alterations in the cell lines NCI‐N87, MKN1 and MKN7. Table S5. Pairwise comparisons of metabolic activity between trastuzumab (T), afatinib (A) and trastuzumab + afatinib (T + A) treated NCI‐N87, MKN1, MKN7 and Hs746T cells. Table S6. Pairwise comparisons of EGFR, MAPK and AKT activation between treatment (afatinib) and control (DMSO) in Hs746T and NCI‐N87 cells. [file MOL2-12-441-s001.pdf]

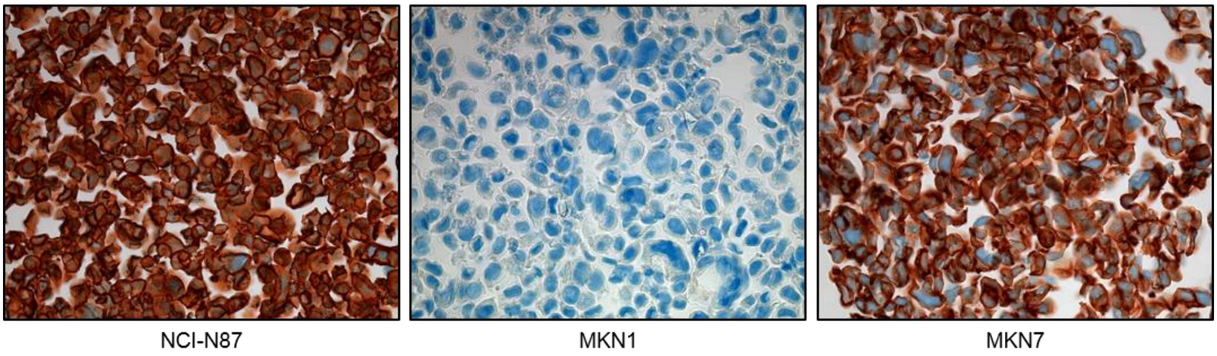

Figure S1 – Assessment of the HER2 status and localization. The HER2 status in the gastric cancer cell lines MKN1, MKN7 and NCI-N87 was evaluated by IHC. The expression of the receptor was analyzed using the HER2-specific antibody 4B5. The staining of HER2 in the cell lines NCI-N87 and MKN7 is positive (score 3+), whereas it is negative (score 0) for MKN1. HER2 is mainly but not exclusively located at the plasma membrane. Pictures were recorded with a 40 x resolution.

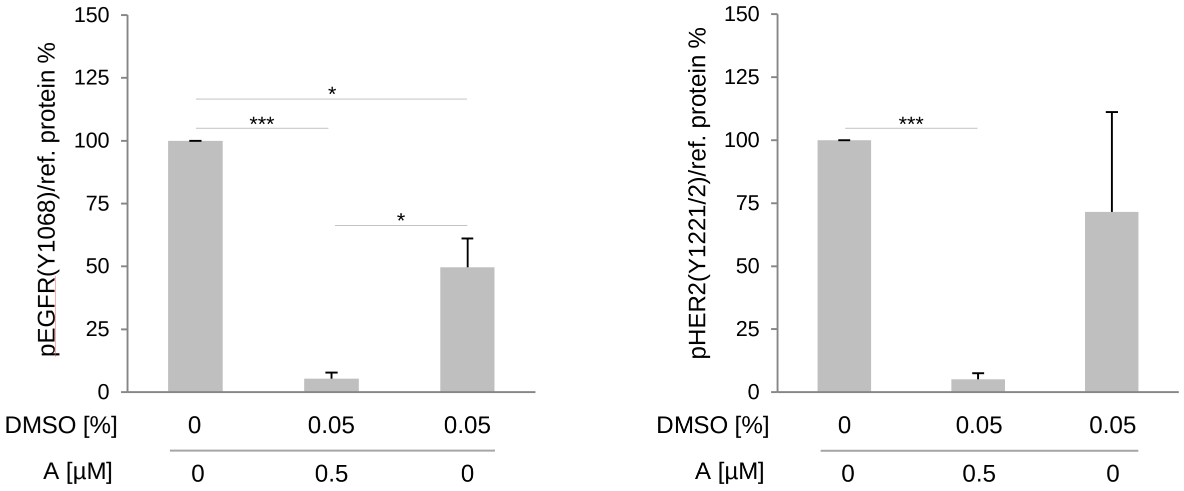

Figure S2 - Effects of DMSO on the activation of EGFR and HER2 in NCI-N87 cells. Protein activation was detected in total lysates of DMSO and afatinib treated NCI-N87 cells by Western blotting using phospho-specific antibodies directed against (a) pEGFR (Y1068) and (b) pHER2 (Y1221/2). A significant (pEGFR) and slight (pHER2) effect of DMSO was detected, but decrease in activation of EGFR and HER2 in afatinib-treated NCI-N87 cells was more pronounced compared to DMSO-controls, thus the detected effects result from afatinib. The mean value of at least three independent experiments is shown. Significant effects are indicated by *$0.01 < p\text{-value} \leq 0.05$ or ***≤ 0.001 (one-sample t-test). Abbreviation: A = afatinib

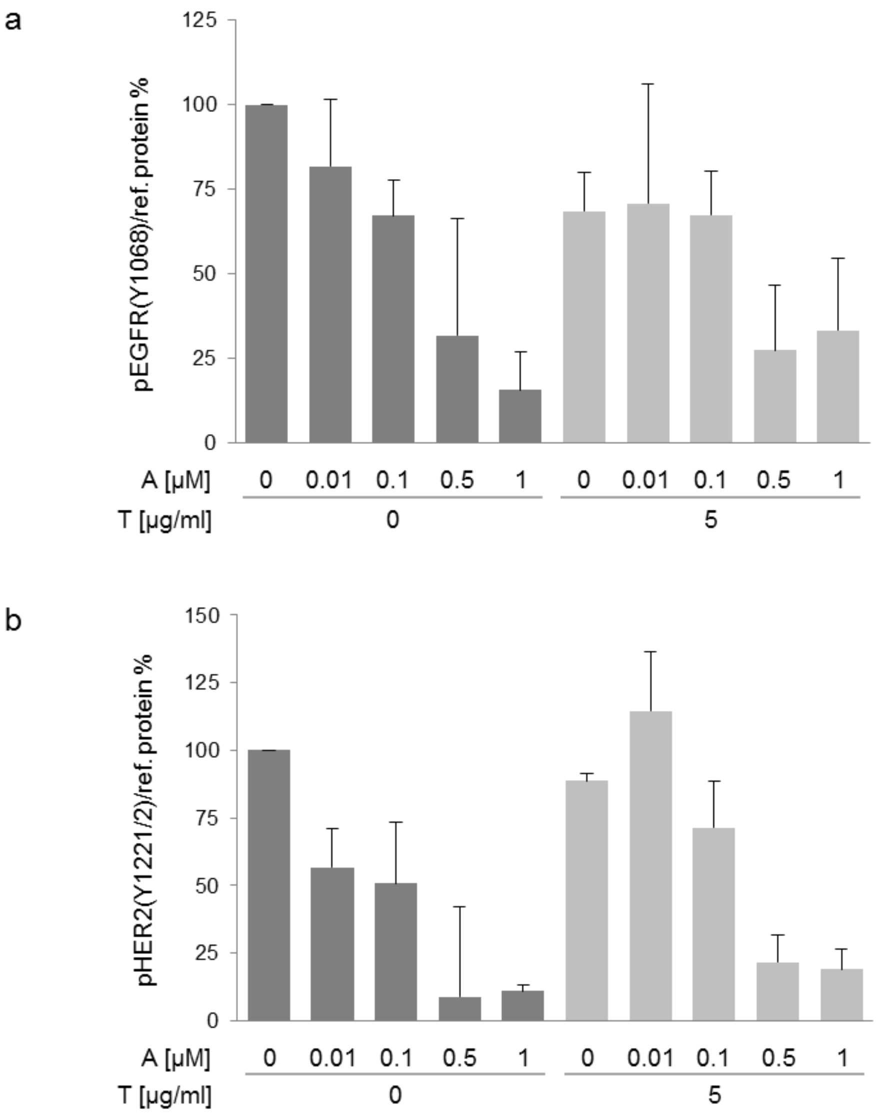

Figure S3 - Concentration-dependent effect of trastuzumab and afatinib on the activation of EGFR and HER2 in NCI-N87 cells. The levels of activated receptors were determined after 5 minute treatment of NCI-N87 cells with trastuzumab (5 µg/ml) and/or afatinib (0.01, 0.1, 0.5, 1 µM) by Western blot analysis with pEGFR-specific (Y1068) and pHER2-specific (Y1221/2) antibodies. The average phosphorylation levels were quantified using densitometric analysis and calculated in relation to the levels of the reference proteins α-tubulin and β-actin (+SD). The mean value of at least three independent experiments is shown. Statistical p-values are listed in Table S1. Abbreviations: A = afatinib, T = trastuzumab

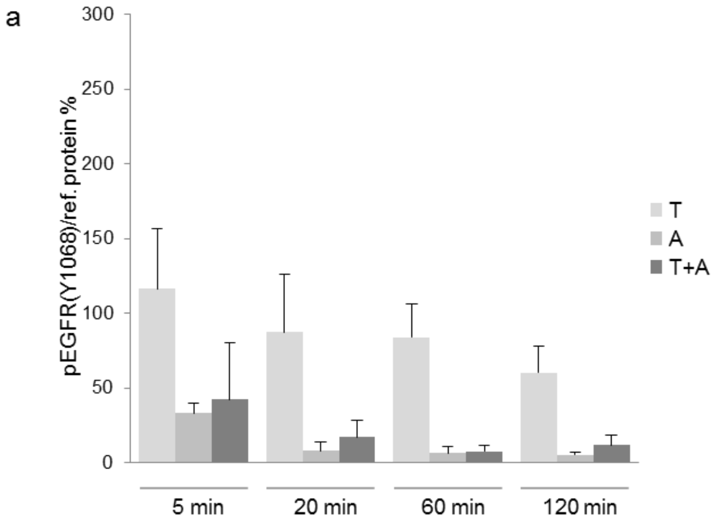

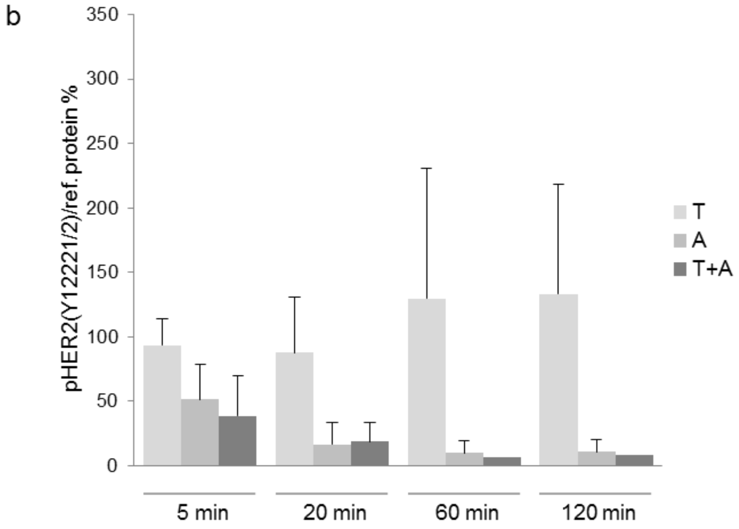

Figure S4 - Kinetic effect of trastuzumab and afatinib on the activation of EGFR and HER2 in NCI-N87 cells. The levels of activated receptors were determined after treatment of NCI-N87 cells with trastuzumab (5 µg/ml) and/or afatinib (0.5 µM) by Western blot analysis. Cells were treated for 5, 20, 60 and 120 min. Activation of the receptors were analyzed using pEGFR-specific (Y1068) and pHER2-specific (Y1221/2) antibodies. The average phosphorylation levels were quantified using densitometric analysis and calculated in relation to the levels of the reference proteins α-tubulin and β-actin (+SD). The mean value of at least three independent experiments is shown. Statistical p-values are listed in Table S1. Abbreviations: A = afatinib, T = trastuzumab

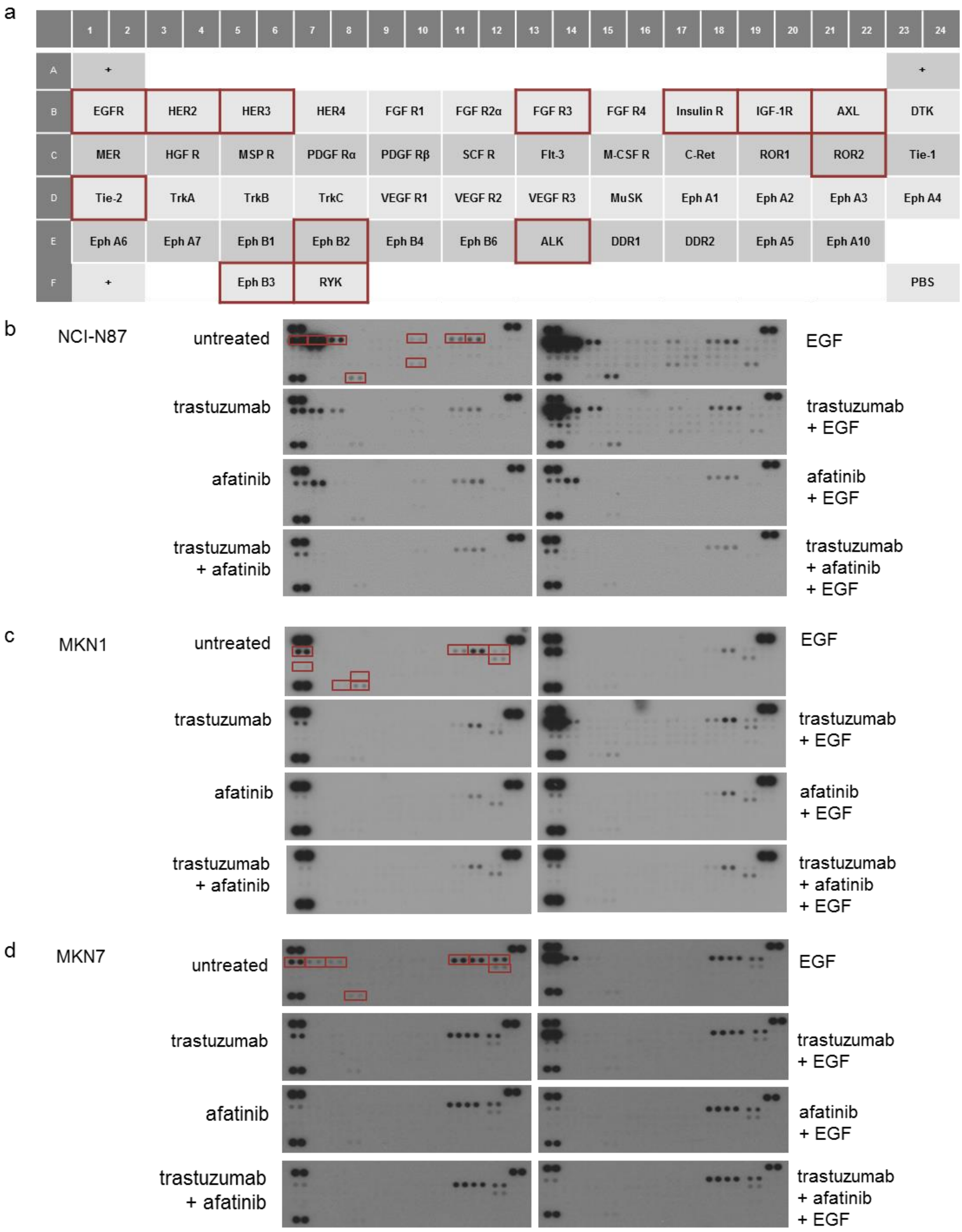

Figure S5 - Receptor tyrosine kinases analyzed by the RTK proteome profiler. (a) Mapped receptor tyrosine kinases on the membrane of the RTK proteome profiler and the entirety of activated proteins (red squares) in the investigated cell lines (+, positive control; PBS, negative control). (b)-(c) Cells were treated for 20 min with 5 µg/ml trastuzumab, 0.5 µM afatinib and/or 5 ng/ml EGF, respectively. Activated, and under different conditions

regulated proteins were detected with chemiluminescence. Varied proteins were activated in the cell line (b) NCI-N87, (c) MKN1 and (d) MKN7 indicated by red squares. The depicted results are representative of three independent experiments.

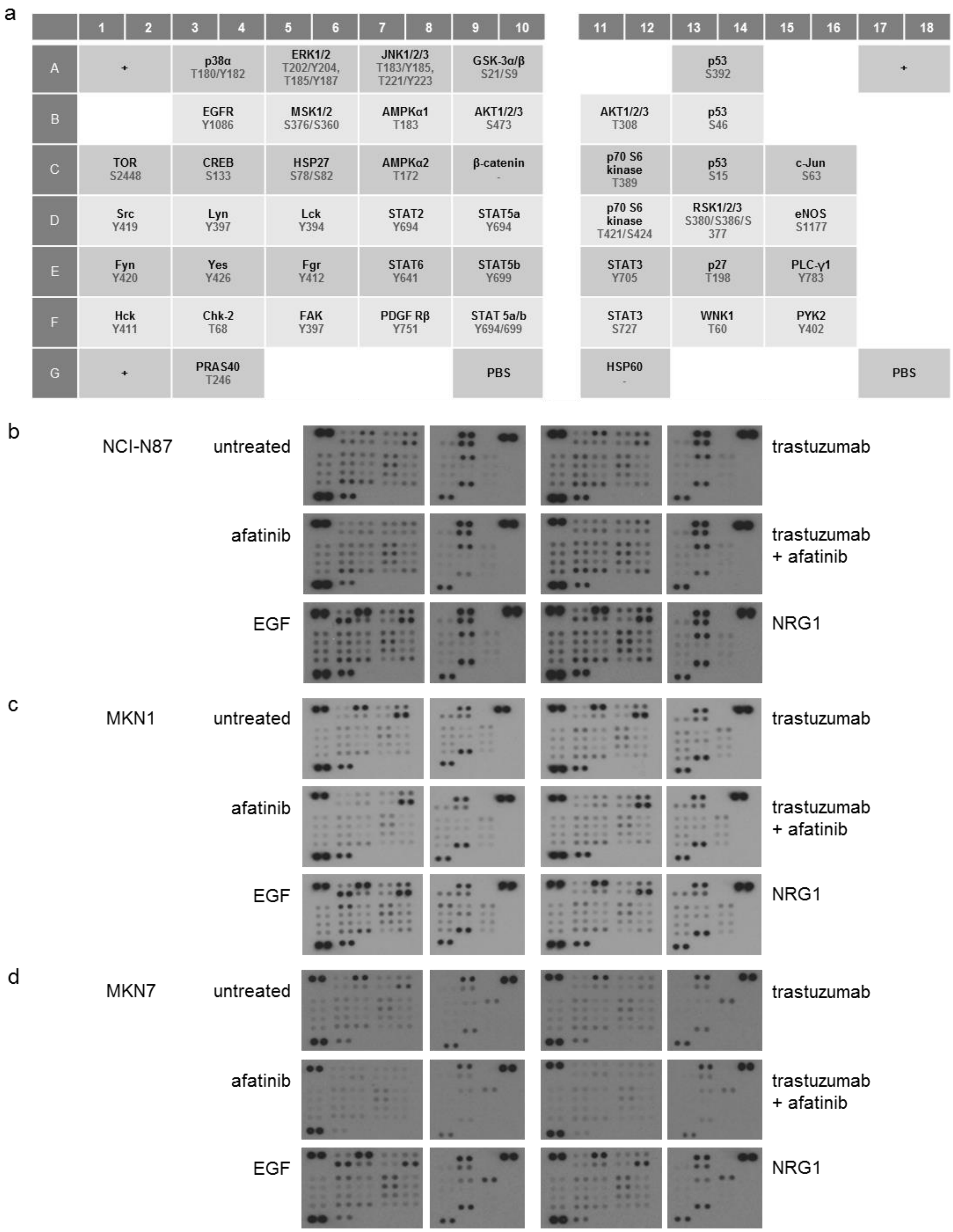

Figure S6 - Phosphorylated kinases analyzed by the kinase proteome profiler. (a) Arrangement of the kinases in the kinase proteome profiler assay, indicating the specific phosphorylation sites. (b)-(c) Cells were treated for 20 min with 5 µg/ml trastuzumab and/or 0.5 µM afatinib or for 5 min with 5ng/ml EGF or 20 ng/ml NRG1, respectively. The depicted results are representative of three independent experiments of the cell lines NCI-N87 (b), MKN1 (c) and MKN7 (d).

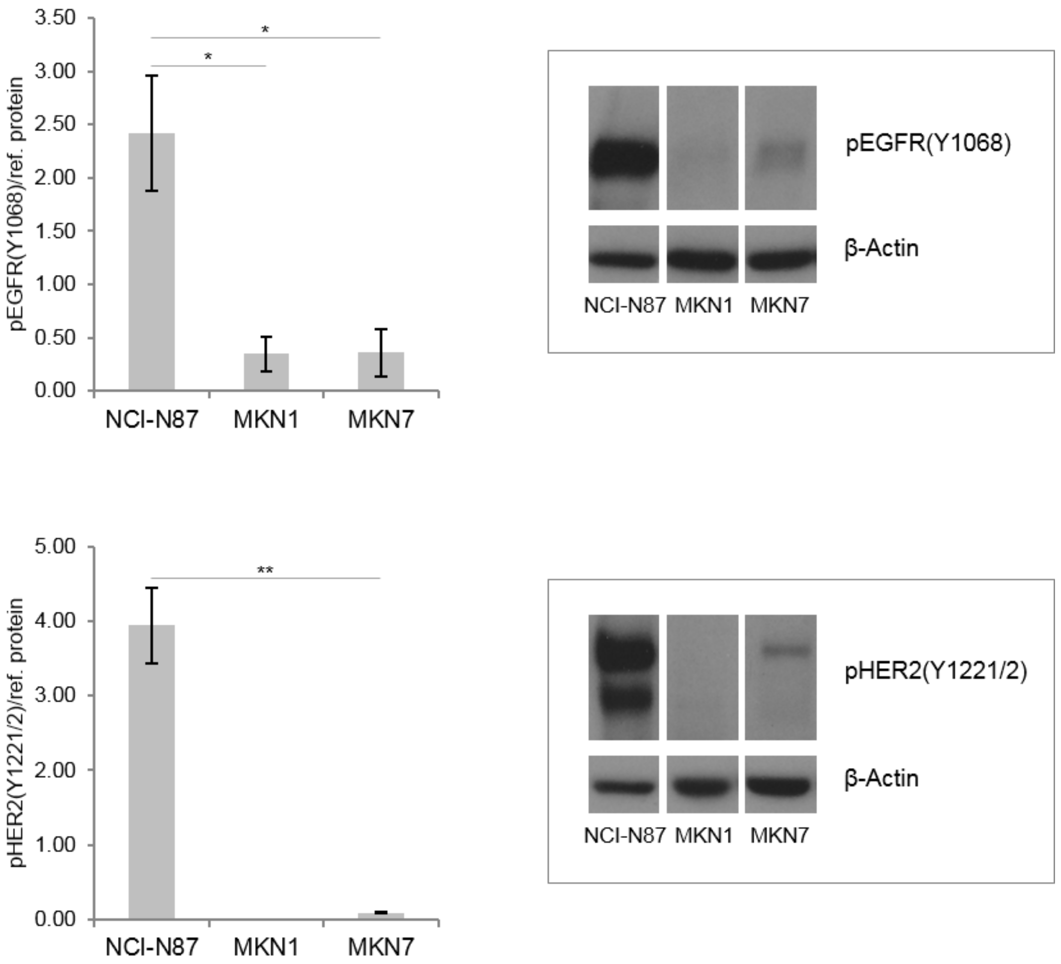

Figure S7 – Comparison of EGFR and HER2 activation in NCI-N87, MKN1 and MKN7 cells. Protein activation was detected in total lysates of untreated cells by Western blotting using phospho-specific antibodies directed against (a) pEGFR (Y1068) and (b) pHER2 (Y1221/2). The average phosphorylation levels were quantified using densitometric analysis and calculated in relation to the levels of the reference proteins α-tubulin and β-actin (+SD). The three different cell lines were detected on one membrane for direct comparison. White lines indicate samples which were cut out since they are not important in this context. Significant effects are indicated by *0.01 < p-value ≤ 0.05 or **0.001 < p-value ≤ 0.01 (Two-sided Welch t-test). No statistical analysis was performed for the activation of HER2 in MKN1 because signals were below detection limits.

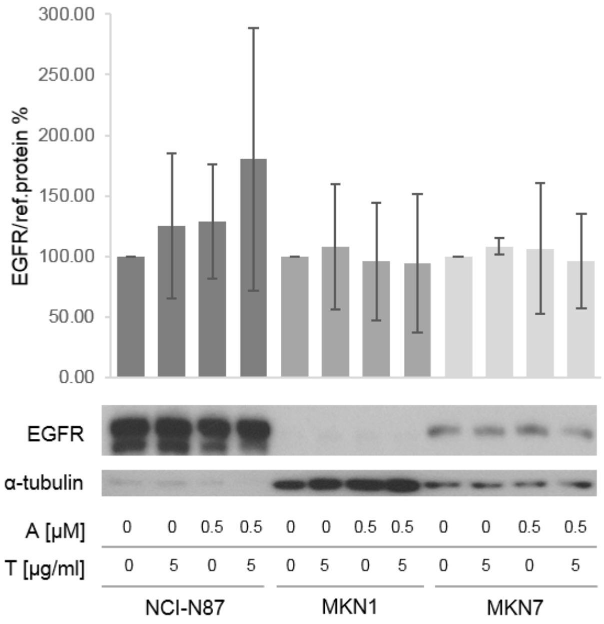

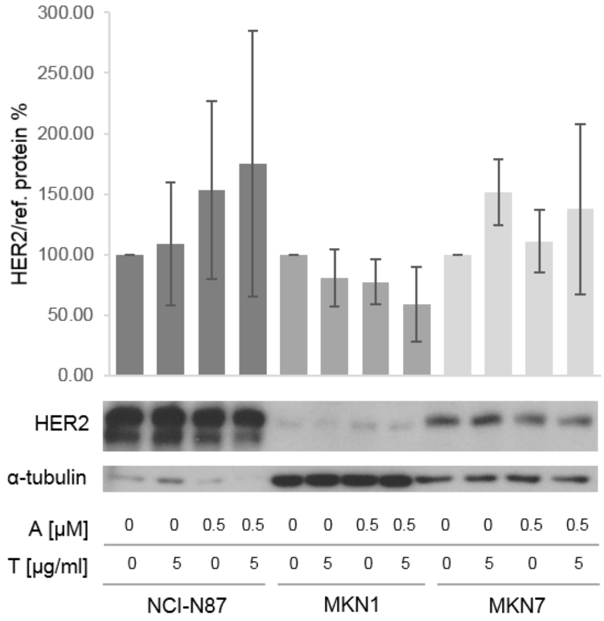

Figure S8 – Effect of trastuzumab and afatinib on the expression of EGFR and HER2 in NCI-N87, MKN1 and MKN7 cells. Protein expression was detected in total lysates by Western blotting using specific antibodies directed against (a) EGFR and (b) HER2. The same lysates were used as generated for the RTK Proteome Profiler. The average abundance levels were quantified using densitometric analysis and calculated in relation to the levels of the reference proteins α-tubulin (+SD). The three different cell lines were detected on one membrane for direct comparison. Quantitative signals are only comparable within one cell line. For each individual cell line, the untreated sample was set to 100 % to visualize the effect of the different treatments. One samples t-test was used to test for statistical significant differences.

Table S1 - Pairwise comparisons of EGFR and HER2 activation in NCI-N87 cells (treatment and control). Cells were treated with afatinib or a corresponding amount of DMSO as control. Two-sided Welch t-test and one-sample t-test were used. Significant p-values are shown. Abbreviations: Ctrl: Control (untreated); A: 0.5 µM afatinib, DMSO: 0.05% DMSO

**Activation of protein**	**Groups (X vs. Y)**				
	**X**	**Y**	**Mean ± SD (X)**	**Mean ± SD (Y)**	**p-value**
**EGFR**	Ctrl	A	100	5.280 ± 2.503	< 0.001
		DMSO	100	49.697 ± 11.368	0.017
	A	DMSO	5.280 ± 2.503	49.697 ± 11.368	0.017
**HER2**	Ctrl	A	100	5.160 ± 2.380	< 0.001

Table S2 - Pairwise comparisons of EGFR, MAPK and AKT activation in NCI-N87 cells between various treatments. Cells were treated with 5 µg/ml trastuzumab and/or different concentrations of afatinib. Western blot analyses were done in biological replicates. A two-sided Welch t-test and a one-sample t-test were used. Significant p-values are shown, relevant comparisons are highlighted in bold font. Abbreviations: Ctrl: Control (untreated); T: 5 µg/ml trastuzumab; A 0.01: 0.01 µM afatinib; A 0.1: 0.1 µM afatinib; A 0.5: 0.5 µM afatinib; A 1: 1 µM afatinib

Activation of protein	Groups (X vs. Y) X	Y	Mean ± SD (X)	Mean ± SD (Y)	p-value
**EGFR**	**Ctrl**	**A 0.1**	**100**	**67.183 ± 10.495**	**0.032**
	**Ctrl**	**A 1**	**100**	**15.766 ± 11.208**	**0.006**
	**Ctrl**	**T**	**100**	**68.626 ± 11.529**	**0.042**
	**Ctrl**	**T+A 0.1**	**100**	**67.487 ± 13.089**	**0.050**
	**Ctrl**	**T+A 0.5**	**100**	**27.565 ± 19.116**	**0.022**
	**Ctrl**	**T+A 1**	**100**	**33.342 ± 21.202**	**0.032**
	**A 0.01**	**A 1**	**81.808 ± 19.764**	**15.766 ± 11.208**	**0.013**
	A 0.01	T+A 0.5	81.808 ± 19.764	27.565 ± 19.116	0.027
	A 0.01	T+A 1	81.808 ± 19.764	33.342 ± 21.202	0.045
	**A 0.1**	**A 1**	**67.183 ± 10.495**	**15.766 ± 11.208**	**0.004**
	A 0.1	T+A 0.5	67.183 ± 10.495	27.565 ± 19.116	0.049
	A 1	T	15.766 ± 11.208	68.626 ± 11.529	0.005
	A 1	T+A 0.1	15.766 ± 11.208	67.487 ± 13.089	0.007
	**T**	**T+A 0.5**	**68.626 ± 11.529**	**27.565 ± 19.116**	**0.044**
	**T+A 0.1**	**T+A 0.5**	**67.487 ± 13.089**	**27.565 ± 19.116**	**0.047**
**HER2**	**Ctrl**	**A 1**	**100**	**10.081 ± 2.085**	**0.000**
	**Ctrl**	**T**	**100**	**88.465 ± 2.556**	**0.016**
	**Ctrl**	**T+A 0.5**	**100**	**22.512 ± 9.852**	**0.005**
	**Ctrl**	**T+A1**	**100**	**21.163 ± 7.513**	**0.003**
	**A 0.01**	**A 1**	**65.003 ± 14.329**	**10.081 ± 2.085**	**0.020**
	A 0.01	T+A 0.5	65.003 ± 14.329	22.512 ± 9.852	0.017
	A 0.01	T+A 1	65.003 ± 14.329	21.163 ± 7.513	0.018
	A 0.5	T+A 0.01	27.935 ± 33.123	105.895 ± 21.821	0.034

**A 1**	**T**	**10.081 ± 2.085**	**88.465 ± 2.556**	**0.000**
A 1	T+A 0.01	10.081 ± 2.085	105.895 ± 21.821	0.016
A 1	T+A 0.1	10.081 ± 2.085	78.367 ± 17.299	0.020
**T**	**T+A 0.5**	**88.465 ± 2.556**	**22.512 ± 9.852**	**0.005**
**T**	**T+A 1**	**88.465 ± 2.556**	**21.163 ± 7.513**	**0.002**
**T+A 0.01**	**T+A 0.5**	**105.895 ± 21.821**	**22.512 ± 9.852**	**0.011**
**T+A 0.01**	**T+A 1**	**105.895 ± 21.821**	**21.163 ± 7.513**	**0.014**
**T+A 0.1**	**T+A 0.5**	**78.367 ± 17.299**	**22.512 ± 9.852**	**0.015**
**T+A 0.1**	**T+A 1**	**78.367 ± 17.299**	**21.163 ± 7.513**	**0.017**

Table S3 - Pairwise comparisons of the EGFR and HER2 activation in NCI-N87 cells between different stimulation times. Cells were treated with 5 µg/ml trastuzumab and/or 0.5 µM afatinib for different stimulation times. Western blot analyses were done in biological replicates. Two-sided Welch t-test and one-sample t-test were used. Significant p-values are shown, relevant comparisons are highlighted in bold. Gray and marked (*) are samples with only two biological replicates. Abbreviations: Ctrl: Control (untreated); T: 5 µg/ml trastuzumab; A: 0.5 µM afatinib; min: minutes

Activation of protein	Groups (X vs. Y) X	Y	Mean ± SD (X)	Mean ± SD (Y)	p-value
**EGFR**	**Ctrl**	**A 5 min**	**100**	**34.544 ± 4.662**	**0.002**
	**Ctrl**	**A 20 min**	**100**	**6.338 ± 7.811**	**0.002**
	**Ctrl**	**A 60 min**	**100**	**4.530 ± 6.146**	**0.001**
	**Ctrl**	**A 120 min**	**100**	**3.858 ± 3.676**	**0.000**
	**Ctrl**	**T+A 20 min**	**100**	**13.841 ± 15.348**	**0.010**
	**Ctrl**	**T+A 60 min**	**100**	**4.619 ± 5.429**	**0.001**
	**Ctrl**	**T+A 120 min**	**100**	**9.028 ± 9.739**	**0.004**
	T 5 min	A 20 min	114.987 ± 41.273	6.338 ± 7.811	0.041
	T 5 min	A 60 min	114.987 ± 41.273	4.530 ± 6.146	0.041
	T 5 min	A 120 min	114.987 ± 41.273	3.858 ± 3.676	0.042
	T 5 min	T+A 20 min	114.987 ± 41.273	13.841 ± 15.348	0.038
	T 5 min	T+A 60 min	114.987 ± 41.273	4.619 ± 5.429	0.042
	T 5 min	T+A 120 min	114.987 ± 41.273	9.028 ± 9.739	0.041
	T 60 min	A 20 min	84.089 ± 22.515	6.338 ± 7.811	0.018
	T 60 min	A 60 min	84.089 ± 22.515	4.530 ± 6.146	0.020
	T 60 min	A 120 min	84.089 ± 22.515	3.858 ± 3.676	0.023
	T 60 min	T+A 20 min	84.089 ± 22.515	13.841 ± 15.348	0.015
	**T 60 min**	**T+A 60 min**	**84.089 ± 22.515**	**4.619 ± 5.429**	**0.021**
	T 60 min	T+A 120 min	84.089 ± 22.515	9.028 ± 9.739	0.017
	T 120 min	A 20 min	61.254 ± 16.818	6.338 ± 7.811	0.017
	T 120 min	A 60 min	61.254 ± 16.818	4.530 ± 6.146	0.018
	**T 120 min**	**A 120 min**	**61.254 ± 16.818**	**3.858 ± 3.676**	**0.023**
	T 120 min	T+A 20 min	61.254 ± 16.818	13.841 ± 15.348	0.023
	T 120 min	T+A 60 min	61.254 ± 16.818	4.619 ± 5.429	0.020

	**T 120 min**	**T+A 120 min**	**61.254 ± 16.818**	**9.028 ± 9.739**	**0.016**
	**A 5 min**	**A 20 min**	**34.544 ± 4.662**	**6.338 ± 7.811**	**0.010**
	**A 5 min**	**A 60 min**	**34.544 ± 4.662**	**4.530 ± 6.146**	**0.003**
	**A 5 min**	**A 120 min**	**34.544 ± 4.662**	**3.858 ± 3.676**	**0.001**
	A 5 min	T+A 60 min	34.544 ± 4.662	4.619 ± 5.429	0.002
	A 5 min	T+A 120 min	34.544 ± 4.662	9.028 ± 9.739	0.029
**HER2**	**Ctrl**	**A 20 min**	**100**	**16.572 ± 17.240**	**0.014**
	**Ctrl**	**A 60 min**	**100**	**9.840 ± 9.892**	**0.004**
	**Ctrl**	**A 120 min**	**100**	**10.874 ± 9.548**	**0.004**
	**Ctrl**	**T+A 20 min**	**100**	**18.713 ± 15.015**	**0.011**
	**Ctrl**	**T+A 60 min***	**100**	**6.415 ± 4.623**	**0.022**
	**Ctrl**	**T+A 120 min***	**100**	**8.772 ± 7.458**	**0.037**
	T 5 min	A 20 min	93.466 ± 20.930	16.572 ± 17.240	0.009
	T 5 min	A 60 min	93.466 ± 20.930	9.840 ± 9.892	0.010
	T 5 min	A 120 min	93.466 ± 20.930	10.874 ± 9.548	0.010
	T 5 min	T+A 20 min	93.466 ± 20.930	18.713 ± 15.015	0.009
	T 5 min	T+A 60 min*	93.466 ± 20.930	6.415 ± 4.623	0.014
	T 5 min	T+A 120 min*	93.466 ± 20.930	8.772 ± 7.458	0.011

Table S4 - Alterations in the cell lines NCI-N87, MKN1 and MKN7. Mutations and types of mutation are listed as well as CNV, Expression level (Z-Score) and Average Ploidy. Data are selected from COSMIC Cell Line Project, canSAR[a] database and Arienti et al.[b] (Forbes et al. 2015, Tym et al. 2016, Arienti et al. 2016). Abbreviations: N/A: not available

**Cell line**	**Mutation** Gene	AA mutation	Type	**CNV** Gene	Expression level (Z-Score)	Copy number	Average ploidy
**NCI-N87**	*ERBB2*	p.F425L[a]	Substitution-*Missense*	*ERBB2*	3.62	14	1.81
		p.S413L[a]	Substitution-Missense				
		p.L436V[a]	Substitution-Missense				
		p.D417D[a]	Substitution-Coding silent				
		p.T900P[b]	Substitution-Missense				
		p.R898G[b]	Substitution-Missense				
		p.Q902K[b]	Substitution-Missense				
		p.R897G[b]	Substitution-Missense				
		p.Q902R[b]	Substitution-Missense				
	CHEK2	p.Q330Q	Substitution-Coding silent				
				GSK3A	2.35	2[a]	1.81
				MAPK14	-3.05	1[a]	1.81
				MTOR	-2.69	N/A	1.81
	PDGFRB	p.P721P	Substitution-Coding silent				
	TP53	p.R248Q	Substitution-Missense				
	TP53	p.R155Q	Substitution-Missense				
**MKN1**				*ERBB2*[a]	N/A	3	2.98
	PIK3CA	*p.E545K*[a]	Substitution-Missense				
				PRKAA1	4.34	5[a]	2.98
	TP53	*p.V143A*	Substitution-Missense				
	TP53	*p.R210H*	Substitution-Missense				
**MKN7**	*EGFR*	p.I923I	Substitution-Coding silent				
				ERBB2	3.19	14	3.02
				JUN	2.23	4[a]	3.02
				PRKAA1	2.58	6[a]	3.02
	TP53	p.278S	Substitution-Missense				
				YES1	2.12	5[a]	3.02

Arienti, C., M. Zanoni, S. Pignatta, A. Del Rio, S. Carloni, M. Tebaldi, G. Tedaldi, and A. Tesei. 2016. "Preclinical evidence of multiple mechanisms underlying trastuzumab resistance in gastric cancer." *Oncotarget* 7 (14):18424-39. doi: 10.18632/oncotarget.7575.

Forbes, S. A., D. Beare, P. Gunasekaran, K. Leung, N. Bindal, H. Boutselakis, M. Ding, S. Bamford, C. Cole, S. Ward, C. Y. Kok, M. Jia, T. De, J. W. Teague, M. R. Stratton, U. McDermott, and P. J.

Campbell. 2015. "COSMIC: exploring the world's knowledge of somatic mutations in human cancer." *Nucleic Acids Res* 43 (Database issue):D805-11. doi: 10.1093/nar/gku1075.
Tym, J. E., C. Mitsopoulos, E. A. Coker, P. Razaz, A. C. Schierz, A. A. Antolin, and B. Al-Lazikani. 2016. "canSAR: an updated cancer research and drug discovery knowledgebase." *Nucleic Acids Res* 44 (D1):D938-43. doi: 10.1093/nar/gkv1030.

Table S5 - Pairwise comparisons of metabolic activity between trastuzumab (T), afatinib (A) and trastuzumab + afatinib (T+A) treated NCI-N87, MKN1, MKN7 and Hs746T cells. Cells were treated with trastuzumab, afatinib, trastuzumab + afatinib, trastuzumab solvent, afatinib solvent or trastuzumab + afatinib solvent. Two-sided Welch t-test and one-sample t-test were used. Significant p-values are shown.

Cell line	Groups (X vs. Y)				
	X	Y	Mean ± SD (X)	Mean ± SD (Y)	p-value
**NCI-N87**	**Ctrl**	**A**	**100**	**35.416 ± 3.966**	**0.001**
	**Ctrl**	**T+A**	**100**	**36.231 ± 4.530**	**0.002**
	T	A	92.041 ± 7.763	35.416 ± 3.966	0.002
	**T**	**T+A**	**92.041 ± 7.763**	**36.231 ± 4.530**	**0.001**
	Solvent T	A	98.007 ± 11.888	35.416 ± 3.966	0.007
	Solvent T	T+A	98.007 ± 11.888	36.231 ± 4.530	0.006
	**A**	**Solvent A**	**35.416 ± 3.966**	**99.941 ± 11.609**	**0.006**
	A	Solvent T+A	35.416 ± 3.966	97.395 ± 15.688	0.016
	Solvent A	T+A	99.941 ± 11.609	36.231 ± 4.530	0.005
	**T+A**	**Solvent T+A**	**36.231 ± 4.530**	**97.395 ± 15.688**	**0.015**
**MKN1**	**Ctrl**	**A**	**100**	**67.078 ± 6.441**	**0.013**
	**Ctrl**	**T+A**	**100**	**68.417 ± 2.066**	**0.001**
	T	A	98.765 ± 3.859	67.078 ± 6.441	0.004
	**T**	**T+A**	**98.765 ± 3.85**	**68.417 ± 2.066**	**0.001**
	Solvent T	A	102.104 ± 5.981	67.078 ± 6.441	0.002
	Solvent T	T+A	102.104 ± 5.981	68.417 ± 2.066	0.006
	**A**	**Solvent A**	**67.078 ± 6.441**	**106.387 ± 7.298**	**0.002**
	A	Solvent T+A	67.078 ± 6.441	103.631 ± 7.403	0.003
	Solvent A	T+A	106.387 ± 7.298	68.417 ± 2.066	0.008
	**T+A**	**Solvent T+A**	**68.417 ± 2.066**	**103.631 ± 7.403**	**0.010**
**MKN7**	**Ctrl**	**T**	**100**	**106.189 ± 1.335**	**0.015**
	**Ctrl**	**Solvent T**	**100**	**107.701 ± 0.979**	**0.005**
	T	A	106.189 ± 1.335	85.796 ± 8.316	0.048
	Solvent T	A	107.701 ± 0.979	85.796 ± 8.316	0.043
	**A**	**Solvent A**	**85.796 ± 8.316**	**108.610 ± 6.179**	**0.022**
	A	Solvent T+A	85.796 ± 8.316	108.311 ± 3.612	0.028

	Solvent A	T+A	108.610 ± 6.179	88.076 ± 8.887	0.036
	**T+A**	**Solvent T+A**	**88.076 ± 8.887**	**108.311 ± 3.612**	**0.044**

Table S6 - Pairwise comparisons of EGFR, MAPK and AKT activation between treatment (afatinib) and control (DMSO) in Hs746T and NCI-N87 cells. Cells were treated with afatinib or corresponding amount of DMSO as control. Two-sided Welch t-test and one-sample t-test were used. Significant p-values are shown. Abbreviations: Ctrl: Control (untreated); A: 0.5 µM afatinib, DMSO: 0.05% DMSO

**Cell line**	**Activation of protein**	**Groups (X vs. Y)**		**Mean ± SD (X)**	**Mean ± SD (Y)**	**p-value**
		**X**	**Y**			
**Hs746T**	**MAPK**	Ctrl	DMSO	100	71.251 ± 7.546	0.022
		A	DMSO	92.809 ±8.584	71.251 ± 7.546	0.032
	**AKT**	Ctrl	A	100	104.468 ± 1.476	0.034
**NCI-N87**	**EGFR**	Ctrl	A	100	5.280 ± 2.503	< 0.001
			DMSO	100	49.697 ± 11.368	0.017
		A	DMSO	5.280 ± 2.503	49.697 ± 11.368	0.017
	**MAPK**	Ctrl	A	100	10.599 ± 5.601	0.001
			DMSO	100	81.846 ± 6.942	0.045
		A	DMSO	10.599 ± 5.601	81.846 ± 6.942	< 0.001
	**AKT**	Ctrl	A	100	10.937 ± 1.516	< 0.001
		A	DMSO	10.937 ± 1.516	128.605 ± 29.990	0.021
